# Study of ß-catenin signaling pathway modulated by PTHrP in cell and animal colorectal cancer models

**DOI:** 10.3389/fonc.2026.1773635

**Published:** 2026-05-29

**Authors:** Cintia Birkenstok, Pedro Carriere, María Belén Novoa Díaz, María Julia Martín, Claudia Gentili

**Affiliations:** 1 Departamento de Biología, Bioquímica y Farmacia, Universidad Nacional del Sur (UNS)- INBIOSUR (CONICET-UNS), Bahía Blanca, Argentina; 2 Departamento de Química, Universidad Nacional del Sur (UNS)- INQUISUR (CONICET-UNS), Bahía Blanca, Argentina

**Keywords:** chemoresistance, colorectal cancer, microenvironment, PTHrP, β-catenin

## Abstract

Colorectal cancer (CRC) is a complex disease and a major cause of cancer-related deaths worldwide. One of the main obstacles in treating advanced CRC is chemotherapy resistance. In previous studies, we demonstrated that parathyroid hormone-related peptide (PTHrP) acts as a pro-tumor cytokine in CRC-derived cells, capable of modulating several mitogenic pathways and inducing β-catenin nuclear localization. Furthermore, we observed that PTHrP favors the communication between tumor cells and their microenvironment, promoting a more aggressive tumor phenotype. Using different CRC models, the first goal of this work was to further explore, how PTHrP is able to modulate the complex β-catenin signaling pathway, whereas the second aim was to examine whether this modulation by PTHrP is associated with resistance to chemotherapy agents commonly used in CRC treatment, particularly platinum-based compounds. The findings obtained *in vitro* by subcellular fractionation, Western blot analysis and immunocytochemistry technique, suggest that PTHrP induces the phosphorylation of β-catenin at Ser552 and its nuclear accumulation. Through viability assays, we found that PTHrP, either directly or indirectly through tumor microenvironment-derived endothelial cells, is associated with reduced oxaliplatin sensitivity in HCT116 cells from CRC. Pharmacological inhibition studies further support the involvement of β-catenin signaling in this effect. *In vivo*, the impact of PTHrP on the oxaliplatin response was model-dependent. In HCT116 xenografts generated in N:NIH(S)_nu mice, in contrast to our *in vitro* findings, oxaliplatin-treated animals showed no significant differences between mice with and without PTHrP within the experimental timeframe. However, we observed that PTHrP reduces oxaliplatin sensitivity in murine CT-26 intestinal tumor cells. In a CT-26 syngeneic murine model, PTHrP was associated with reduced oxaliplatin efficacy and increased β-catenin immunoreactivity. Overall, our findings provide consistent *in vitro* evidence supporting an association between PTHrP, β-catenin signaling, and decreased oxaliplatin sensitivity in CRC cells. However, *in vivo* results suggest a more complex and context-dependent role, highlighting the need for further studies to clarify the mechanistic contribution of β-catenin and the extent to which PTHrP drives chemoresistance in diverse settings.

## 1 Introduction

Colorectal cancer (CRC) is one of the most common tumors of the digestive system, representing the third most prevalent type of malignant neoplasia and the second cause of cancer death worldwide (1). According to the International Agency for Research on Cancer, by 2040 the burden of CRC will increase to 3.2 million new cases per year (an increase of 63%) and 1.6 million deaths per year (an increase of 73%). Due to the largely effectiveness of screening programs, CRC incidence rates have declined in developed countries; however, they have increased in young adults and in developing countries (2).

In recent decades, not only screening strategies have improved, but also CRC treatments, reducing the rate of relapses/recurrences by up to 65–88% (3). Currently, the treatment of this disease mainly consists of surgical resection, immunotherapy, radiotherapy and chemotherapy; in this sense, the most commonly used chemotherapeutic drugs are 5-fluorouracil (5-FU), oxaliplatin, and irinotecan (CPT-11) (4). With regard to oxaliplatin, the chemotherapeutic agent studied in this work, it is a third-generation platinum (Pt)-based compound used as first-line treatment for advanced CRC and as adjuvant therapy following complete primary tumor resection. It exerts its antitumor effects by forming DNA–Pt complex that disrupt replication and transcription, leading to DNA damage and apoptosis (5). Although chemotherapy has improved the overall survival of advanced CRC patients (4), recurrence and metastasis remain frequent due to the development of therapy-resistant CRC subtypes (6).

Over the past decade, the tumor microenvironment (TM) has emerged as a key regulator of CRC progression. The TM comprises the entire cellular milieu surrounding tumor cells, including endothelial cells, immune cells, fibroblasts, mesenchymal cells and the extracellular matrix (7). These cells communicate bidirectionally through autocrine and paracrine factors including cytokines and growth factors, shaping tumor cells behavior (8).

PTHrP is a cytokine whose mode of action is mainly autocrine and paracrine, and it is widely expressed in normal tissues (9, 10) and overexpressed in several cancer types, including CRC (11), where it may be associated with poor prognosis (12, 13). We previously observed that in CRC-derived cells, PTHrP promotes proliferation, cell cycle progression, migration, tumor-related angiogenesis, epithelial to mesenchymal transition (EMT) program, cancer stem cell (CSC) phenotype and resistance to drugs such as CPT-11 and oxaliplatin. PTHrP also favors the communication between tumor cells and HMEC-1 endothelial cells from the TM, thereby promoting a more aggressive CRC behavior. *In vivo*, the cytokine induces morphological changes compatible with the EMT program and neoangiogenesis and modulates the expression of several markers associated with tumor progression events. (14–21).

The Wnt/β-catenin signaling pathway is a central regulator of development and maintenance (22). In CRC this pathway is altered in ~ 90% of cases (23), leading to proliferation, invasion, metastasis and chemotherapy resistance (24). β-catenin normal levels in the cytosol are regulated by a degradation complex formed by the scaffold Axin, the adenomatous polyposis coli (APC) gene product, casein kinase 1 (CK1), and glycogen synthase kinase 3 (GSK3). In absence of Wnt ligand, β-catenin is sequentially phosphorylated at Ser45 by CK1 and at Thr41/Ser37/Ser33 by GSK3 (ubiquitin signal), leading to its proteasomal degradation. By contrast, Wnt binding to its receptors inhibits the action of the degradation complex, resulting in β-catenin into the nucleus where it stimulates the transcription of genes that regulate cell proliferation, differentiation, and migration. The prevalent defects of the described cascade in CRC mutations are in APC, axin, and/or β-catenin and are associated with poor outcome in CRC patients, resulting in exacerbate β-catenin nuclear accumulation (25). Additionally, phosphorylation at other sites such as Ser552 and Ser675 can enhance β-catenin nuclear localization and transcriptional activity independently of canonical Wnt signaling and can also be deregulated in cancer (26). Previously, we found that PTHrP promotes the nuclear localization of β-catenin in CRC-derived Caco-2 cells (21). However, prior to this study, the molecular mechanisms by which PTHrP modulates these phenomena in CRC cells, and whether β-catenin pathway contributes to PTHrP-induced aggressive phenotype, remained unclear.

In light of the findings of our research group over the last 10 years, discussed above, and the importance of β-catenin signaling pathway in CRC, the first goal of this work was to further explore how PTHrP modulates this complex pathway, using *in vitro* and *in vivo* CRC models. The second aim was to examine whether this modulation by PTHrP is associated with resistance to chemotherapy agents commonly used in CRC treatment, particularly platinum-based compounds.

## 2 Materials and methods

### 2.1 Cell lines and culture conditions

For *in vitro* studies, we used Caco-2 cells and HCT116 cells, both derived from human CRC, and murine CT-26 intestinal tumor cells derived from BALB/c mice. All the cell lines were obtained from the ATCC. Human cell lines were cultured in Dulbecco’s Modified Eagle Medium (DMEM), whereas CT-26 cells were cultured in Roswell Park Memorial Institute 1640 (RPMI). All cell lines were maintained in a medium supplemented with 10% fetal bovine serum (FBS), penicillin and streptomycin at 37 °C in a humidified atmosphere containing 5% CO_2_ in air. To start the experiments, Caco-2 cells with 70-80% confluence were deprived of FBS for 24 hours, while the other two cell lines with the same confluence were deprived of FBS for 2 hours, to synchronize the cells in quiescent G0/G1 phase and obtain reproducible results. These times were selected according to our previous studies (17, 19–21). Cells were treated with PTHrP (1-34) (Sigma-Aldrich Chemical Co., St. Louis, Missouri, USA) prepared in DMEM supplemented with 1% FBS at a concentration of 10_–8_ M as we previously reported (19, 21), or with endothelial conditioned media (CM). Where indicated, cells were pre-incubated for 30 minutes with iCRT14 (50 µM), a specific inhibitor of β-catenin transcriptional activity. Control conditions were performed by adding an equivalent volume of dimethylsulfoxide (DMSO<0,1%), the vehicle of the inhibitor. Oxaliplatin was kindly provided by Dr. Ariel Zwenger. HCT116 cells or CT-26 cells were treated with the drug (10 µM or 20 and 50 µM, respectively) for 24 hours. The doses and incubation times of both, the chemotherapeutic drug and the β-catenin inhibitor, were selected according to previous studies (14, 27).

### 2.2 Endothelial CM preparation

As a cellular model of the TM, immortalized human endothelial cells (HMEC-1, from ATCC) were employed. CM was obtained from HMEC-1 cell cultures, treated for 16 hours in the presence or absence of 10–^8^ M PTHrP, according to a previously used protocol (17). CM from control HMEC-1 cells (CCM) and PTHrP-treated HMEC-1 cells (TCM) were collected and centrifuged for 10 minutes at 10,000 rpm to remove cell debris. The supernatants were stored at -80 °C until use for the treatment of HCT116 cells. Each experiment was performed using independently obtained CM.

### 2.3 Subcellular fractionation

The two subcellular fractions were obtained by centrifugation using a refrigerated centrifuge (SIGMA 3K 30). The culture medium was removed, and the cells were washed twice with cold phosphate-buffered saline solution (PBS) and collected in cold TES buffer (50 mM Tris-HCl, 1 mM EDTA, 250 mM sucrose containing protease inhibitors, pH 7.4) for cold homogenization using a glass-Teflon hand homogenizer (30 strokes). The homogenate was then centrifuged at 1200 x g for 20 minutes at 4 °C, and the pellet (nucleus-enriched fraction) was washed twice with a cold TES buffer and finally suspended in the same cold buffer. The supernatant was the post-nuclear fraction.

### 2.4 Western blot analysis

The quantification of the proteins from nuclear fraction, post-nuclear supernatant or from whole cell lysates was performed by the Bradford colorimetric method (28). Denatured proteins present in the samples (30 µg/lane) were separated by electrophoresis on discontinuous SDS-PAGE gels (stacking gel: 4% acrylamide, separating gel: 10% acrylamide) according to the Laemmli technique (29). Colored markers of known molecular weight were seeded concurrently. Electrophoresis was performed at constant voltage (100 volts) using Tris 25 mM pH 8.8; glycine 195 mM; SDS 0.1% as running buffer. Then, the proteins were electrotransferred to hydrophilic polyvinylidene fluoride (PVDF) membranes. The membranes were blocked with 5% skim milk in Tris buffered saline-Tween (TBS-T) buffer (50 mM Tris, pH 7.2-7.4, 200 mM NaCl, 0.1% Tween-20), and after incubated over-night with one of the following primary antibodies, which were diluted in TBS-T with 2.5% bovine serum albumin (BSA): anti-p-β-catenin (Ser552), anti-β-catenin, anti-GAPDH (Cell Signaling Technology, Beverly, Massachusetts, USA), anti-lamin B, anti-cytochrome C (Santa Cruz Biotechnology, Dallas, Texas, USA) or anti-actin (Sigma-Aldrich Chemical Co., St. Louis, Missouri, USA). After washing, the membranes were incubated with the appropriate dilution of horseradish peroxidase conjugated secondary antibody in TBS-T with 2.5% skim milk. Finally, proteins were revealed using a commercial electrochemiluminescence kit, the bands obtained were digitized densitometrically and quantified by the Image J program.

### 2.5 Stripping and reprobe membranes

To remove primary and secondary antibodies, membranes were incubated in stripping buffer (62.5 mM Tris–HCl pH 6.8, 2% SDS and 50 mM β-mercaptoethanol) at 55 °C for 30 minutes in agitation, washed for 10 minutes in TBS-T (1% Tween-20) and then they were blocked, as mentioned above. After this procedure, it was possible to re-test antibodies in the membranes.

### 2.6 Immunocytochemistry

To obtain information of β-catenin subcellular localization, CRC cells seeded on coverslips were fixed and permeabilized with methanol for 15 minutes at -20 °C. After washing with 1X PBS, nonspecific protein-binding sites were blocked with 5% BSA in PBS for 1 hour at room temperature. The samples were then incubated overnight at 4 °C in the presence or absence (negative control) of anti-β-catenin antibody (Fisher Scientific S.L., Spain). After washing with PBS, the preparations were incubated with the corresponding secondary antibody conjugated to the fluorophore Cy3 #A10521 (Life Technologies, California, USA) for 1 hour at room temperature in the dark. Finally, the preparations were mounted with a 95% glycerol solution in PBS, and images were obtained using a NIKON TE 2000S fluorescence microscope. The images were then analyzed using the NIH Image J software, and the corrected total cell fluorescence (CTCF) was quantified according to the following formula: CTCF = Integrated Density – (Cell Area x Average Background Fluorescence) (30).

### 2.7 Measurement of the number of live cells by trypan blue dye exclusion test

After treatment, the cells were trypsinized and stained with a 0.4% solution of trypan blue dye. The cells were visualized using a microscope and a Neubauer chamber, and the viable cells that excluded the dye were counted. The results are expressed as a percentage relative to control.

### 2.8 Measurement of the number of live cells by neutral red uptake assay

The altered lysosomal metabolism by oxaliplatin cytotoxicity was measured using Neutral Red Uptake (NRU) assay (Santa Cruz Biotechnology, Santa Cruz, Dallas, Texas, USA) to evaluate the number of viable HCT116 cells. After treatment, cells were washed with PBS before and after exposure to 0.010% NR solution for 2 hours at 37 °C in a humid atmosphere of 5.5% CO_2_ in air. Ethanol/water/acetic acid (50:49:1) solution was used to dissolve the NR-stained cells and color intensity was measured at 540 nm in a microplate reader (BioTek Synergy-HT). The results were expressed as the percentage of viable cells compared to the control.

### 2.9 HCT116 cells xenografts in nude mice

**Figure 1:**
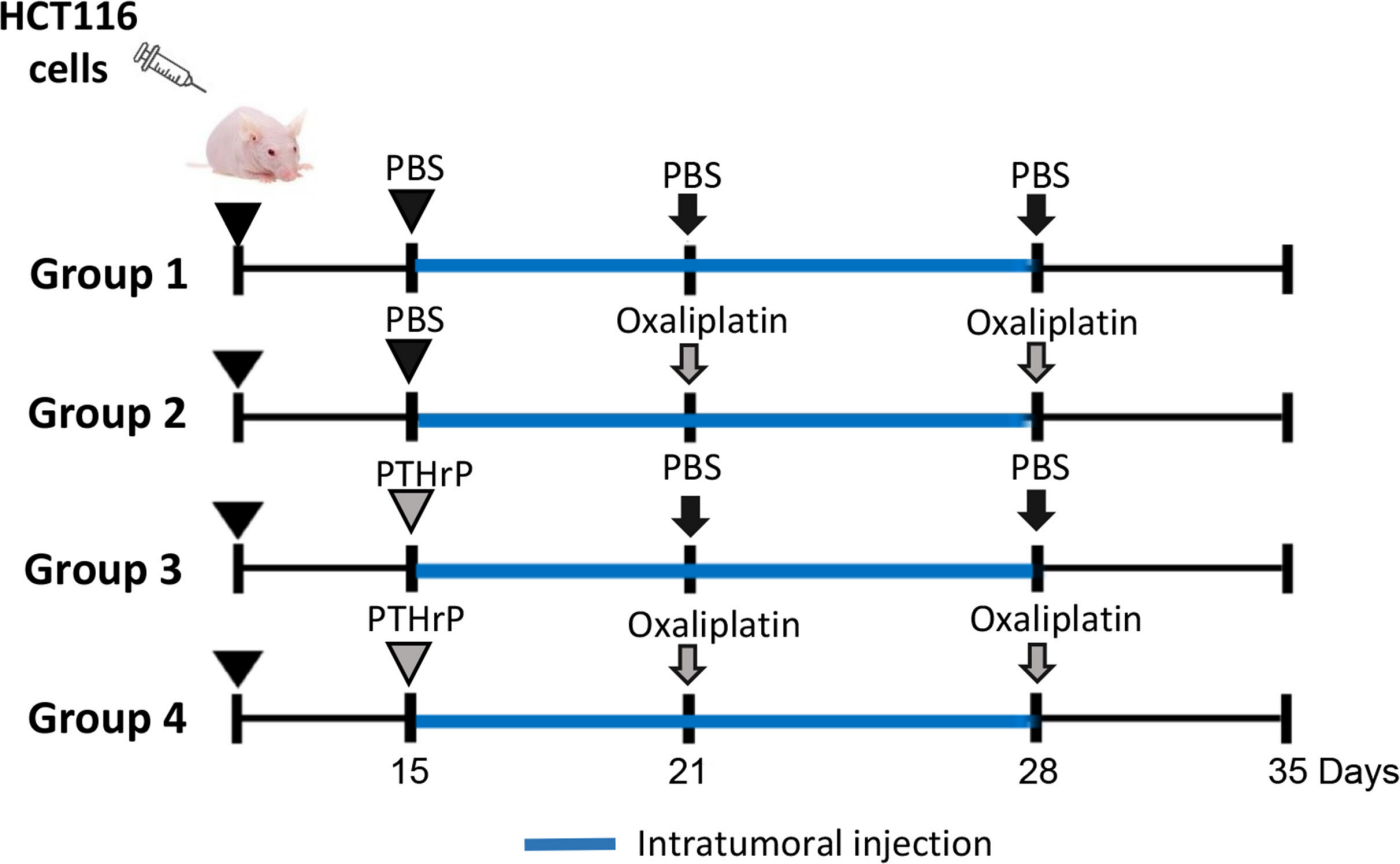
Experimental design of HCT116 cell xenografts in athymic mice. Representation of the protocol used to evaluate the effect of PTHrP on the response to oxaliplatin. HCT116 cells were inoculated subcutaneously into athymic mice. Two weeks after tumor cell implantation, the mice were treated with PTHrP (40 µg/kg) or its corresponding vehicle. Subsequently, the animals received two doses of oxaliplatin (5 mg/kg) according to the indicated treatment regimen. Tumor growth was monitored throughout the experimental period. PBS: phosphate-buffered saline solution. PTHrP: parathyroid hormone-related peptide.

HCT116 cells (1 million in 100 µl of PBS per animal) were injected subcutaneously in the right flank of twenty-threemale N:NIH (S)_nu mice (body weight 20–25 g), according to the protocol described by Dunn and colleagues (31). As shown in **Figure 1**, two weeks later, mice were randomly assigned to four experimental groups: Group 1 (n = 6), treated with 100 μL PBS (PTHrP vehicle); Group 2 (n = 6), treated with PBS plus oxaliplatin (5 mg/kg); Group 3 (n = 6), treated with PTHrP (40 μg/kg, in 100 μL PBS); and Group 4 (n = 5), treated with PTHrP (40 μg/kg) in combination with oxaliplatin (5 mg/kg). PTHrP or vehicle was administered intratumorally for 15 consecutive days. The daily treatment was intended to maintain blood levels of the compound. When the tumors reached a volume of 100 mm³, administration of the cytostatic agent or its vehicle was initiated via intraperitoneal injection (on days 21 and 28 after inoculation of the tumor cells). The dose and number of applications of the chemotherapeutic agent were selected according to previous protocols (32, 33). Tumor size was measured three times a week, and mouse weight was used as an indicator of animal welfare. Animals were euthanized using the chemical agent CO_2_. The euthanasia method was chosen according to the Guidelines for the Care and Use of Laboratory Animals, Institute of Laboratory Animal Resources, Commission on Life Sciences, National Research Council. The tumor growth rate was calculated at the end of the experiment, comparing tumor volume values in the treated groups to those in the control group. All tumors were processed and embedded in paraffin blocks. The experimental protocol was approved by the Institutional Committee for the Care and Use of Experimental Animals (CICUAE) of the National University of the South-UNS (Protocol 237/2024, Registration No. CDBByF 0352/24).

### 2.10 Syngeneic tumor model in Balb/c mice

**Figure 2:**
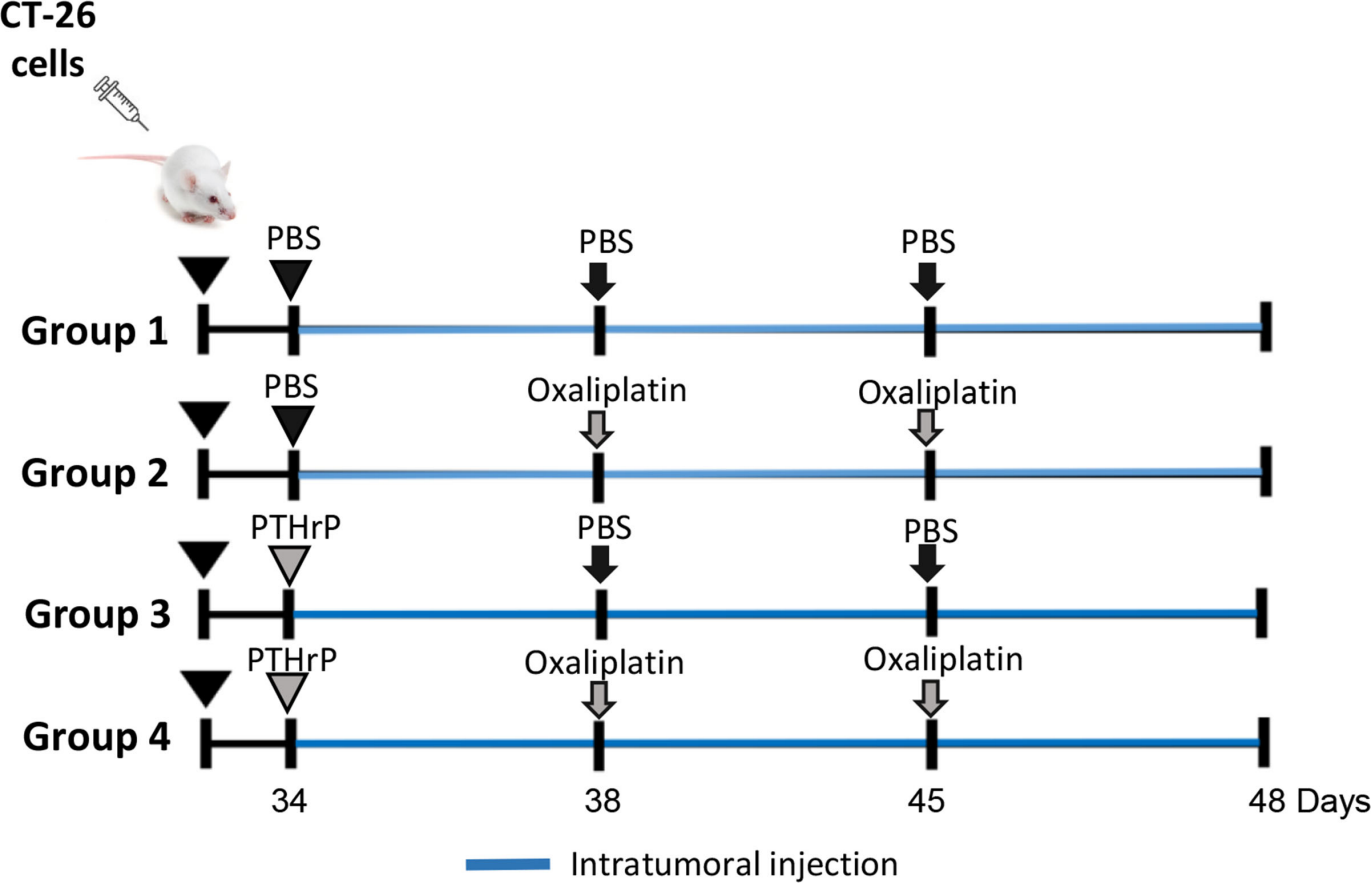
Experimental design of the syngeneic CT-26 tumor model in Balb/c mice. Balb/c mice were subcutaneously inoculated with CT-26 cells and randomly assigned to receive PTHrP (40 µg/kg) or its corresponding vehicle for 14 consecutive days. Subsequently, mice were treated with oxaliplatin (5 mg/kg) or its corresponding vehicle, administered once per week according to the schedule indicated. Tumor growth and animal body weight were monitored throughout the experimental period. PBS, phosphate-buffered saline solution; PTHrP, parathyroid hormone-related peptide.

CT-26 cells (200,000 cells in 100 µl of PBS per animal) were injected into the right flank of twenty-five immunocompetent Balb/c mice, 6 to 8 weeks old (body weight 19–27 g) according to the protocol by Ripoll and collaborators (34). The animals were housed in cages with free access to food and water and were treated in accordance with the ethical principles of animal experimentation. The protocol was approved by CICUAE-UNS (Protocol 236/2024, Registration No. CDBByF 0352/24). When the tumor was detected by palpation (at day 21 post-inoculation), mice were randomly assigned to four experimental groups: Group 1 (n= 6), treated with 100 μL of PBS (PTHrP vehicle), Group 2 (n= 6), treated with 100 μL of PBS plus oxaliplatin (5 mg/kg), Group 3 (n= 7), treated with PTHrP (40 μg/kg in 100 μL PBS), Group 4 (n=6), treated with PTHrP (40 μg/kg in 100 μL PBS) in combination with oxaliplatin (5 mg/kg) (see **Figure 2**). Daily intratumoral administration of either PBS (control) or PTHrP (treated) began on day 34, when the tumors had reached an adequate volume. Four days after peptide or PBS injections, mice received intraperitoneally oxaliplatin or the drug vehicle (PBS) for two weeks (once per week). The dose and number of administrations of the chemotherapeutic agent were selected based on the literature (35). Tumor size was determined three times a week, and the volume was calculated as a x b²/2, where *a* is the largest diameter and *b* is the smallest diameter. Mouse weight was also assessed as an animal welfare parameter, and the tumor growth rate (TGR) was calculated (36). Mice were euthanized by cervical dislocation when tumor volume was appropriate or in cases of significant body weight loss (>10%), tumor ulceration, or other signs of declining health, following FELASA guidelines. Necropsies were then performed to obtain tumors, which were preserved in formalin until paraffin block embedding for subsequent histological evaluation. Additionally, to investigate the presence of metastasis, the right lung, liver and spleen were removed and also processed for paraffin embedding.

### 2.11 Hematoxylin and eosin staining

For visualization of the structural organization of mice tissues, sections of tumor pieces and extracted organs (which were embedded in paraffin) were cut, deparaffinized in xylene and then were hydrated with ethyl alcohol solutions of decreasing concentrations to proceed with H&E staining followed by dehydration for mounting, which allowed the visualization of the stained tissues using an optical microscope.

### 2.12 Immunohistochemistry

Paraffin-embedded tissue sections were cut and prepared for the evaluation of protein levels of the marker by IHC following the same protocol as in our previous studies (14, 16–18, 20). Anti-β-catenin antibody (Cell Signaling Technology, Beverly, Massachusetts, USA) was used as the primary antibody. IHC staining was performed manually using the ABCAM IHC detection kit (Abcam, Cambridge, MA, USA) according to the manufacturer’s instructions. Representative images were obtained using a light microscope and used to quantify optical density by the FIJI plugin of ImageJ-NIH software.

### 2.13 Statistical analysis

Statistical analyses were performed using GraphPad Prism 8.0.2 (San Diego, CA, USA). All *in vitro* experiments were independently repeated at least three times, unless otherwise indicated, and were considered as biological replicates. Technical replicates were included according to the specific assay: trypan blue exclusion assays were performed in triplicate, NRU assays in quintuplicate, and Western blot densitometric analyses were obtained from independent experiments using ImageJ software. For immunocytochemistry analyses, CTCF was quantified from ten random microscopic fields from each experiment. Data are expressed as mean ± standard deviation (SD). For comparisons between two experimental groups, Student’s t-test was used. Two-way ANOVA followed by Tukey’s *post hoc* test were used when two independent variables were analyzed simultaneously or treatment combinations involving PTHrP, oxaliplatin, conditioned media, and/or iCRT14. Sample size in murine models was determined based on previous studies using similar CRC murine models and expected treatment effects. Tumor growth rate (TGR) was calculated as:


TGR(%)=(Vf−Vi)/Vi×100

For histological and immunohistochemical analyses, ten random microscopic fields per section were analyzed from three independent tumor sections per animal using FIJI/ImageJ-NIH software. Optical density quantification and mitotic cell counting were performed under the same acquisition parameters for all groups. A p-value< 0.05 was considered statistically significant, using the following notation: p< 0.05 (*), p< 0.01 (**), and p< 0.001 (***).

## 3 Results

### 3.1 Molecular mechanisms associated with β-catenin signaling pathway modulation in PTHrP-treated CRC cells

**Figure 3:**
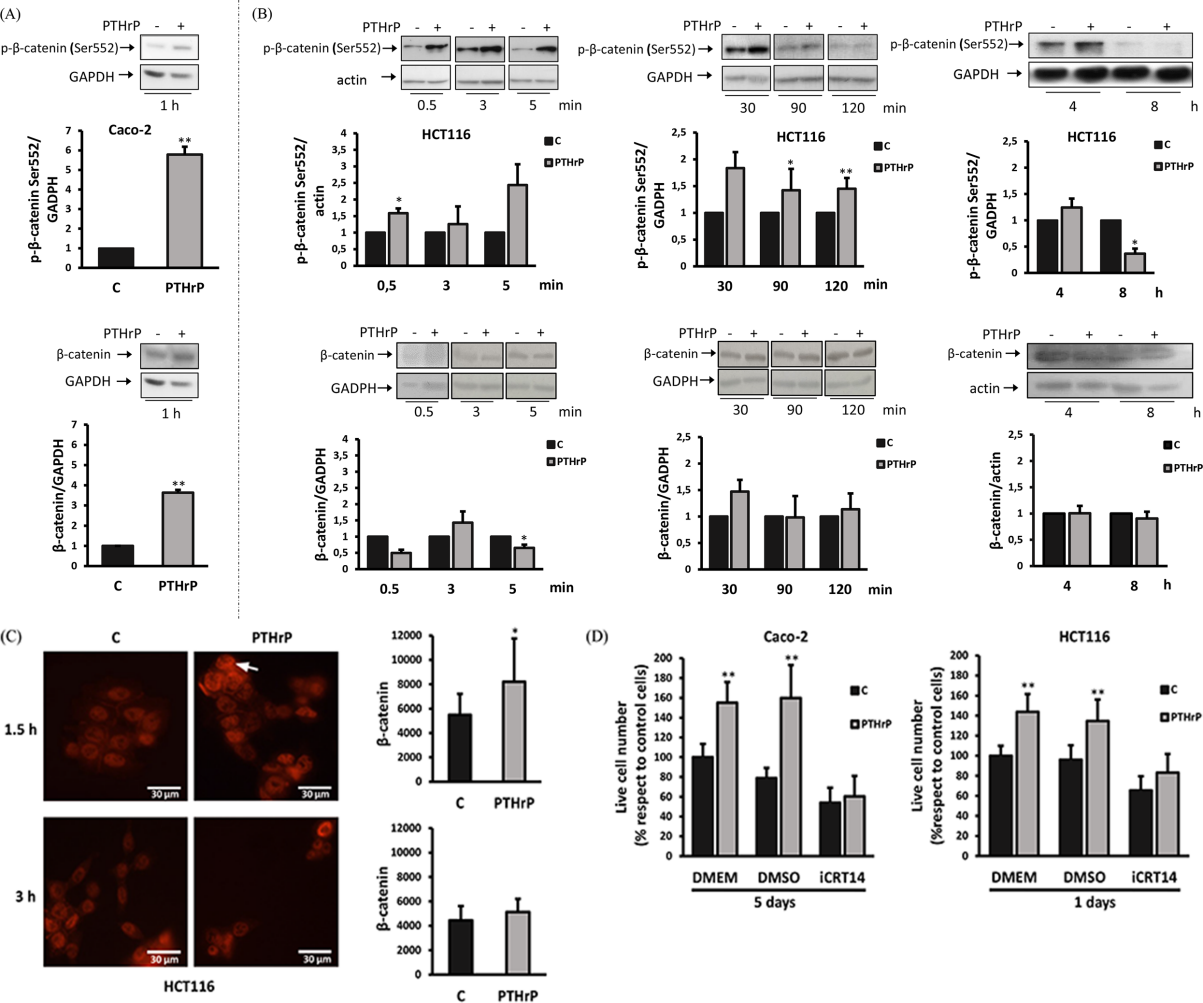
Molecular mechanisms triggered by PTHrP in CRC cells leading to nuclear β-catenin localization and its Wnt-independent activation. **(A)** Caco-2 cells were treated with PTHrP (10^-8^ M) or its vehicle for 1 hour, and β-catenin phosphorylation at Ser552 was assessed by Western blot analysis. GAPDH was used as a loading control. **(B)** HCT116 cells were exposed to PTHrP (10^-8^ M) for the indicated times, and β-catenin phosphorylation levels at Ser552 were analyzed by Western blot analysis, using GAPDH and actin as loading controls. **(C)** subcellular localization of β-catenin was assessed by immunocytochemistry technique in HCT116 cells treated with PTHrP for 90 minutes or 3 hours. The arrow indicates an increase in nuclear accumulation of β-catenin after 90 minutes of treatment. Scale bar = 30 µm. **(D)** Caco-2 and HCT116 cells were pretreated with the β-catenin-responsive transcription inhibitor iCRT14 or its vehicle (DMSO) and subsequently exposed to PTHrP (10^-8^ M) for 5 days or 24 hours, respectively. Trypan blue dye was used to evaluate the number of viable cells. Data are expressed as mean ± SD. For western blot analysis, statistical significance was determined from two independent biological replicates (n=2). For immunocytochemistry analyses, CTCF was quantified from ten random microscopic fields. When using the trypan blue dye exclusion technique, statistical significance was determined from two independent biological replicates (n=2), three technical replicates each. *p< 0.05; **p< 0.01; ***p< 0.001. DMSO: dimethylsulfoxide. PTHrP: parathyroid hormone-related peptide.

β-catenin nuclear accumulation is an event directly associated with CRC genesis and progression (37–39). In Caco-2 cells, we observed that PTHrP favors the nuclear translocation of β-catenin and also increases its target protein levels, c-Myc and Cyclin D (15, 21, 40, 41). Besides, we reported that PTHrP actives Proto-oncogene tyrosine-protein kinase Src (Src), Protein Kinase B (PKB/Akt) and Mitogen-Activated Protein Kinases (MAPKs) signaling pathways in Caco-2 cells and also in HCT116 cells (15, 19–21). These kinases stabilize β-catenin and promote its nuclear accumulation via Wnt-independent phosphorylation at Ser552 (42–44). In view of this background, herein we first investigated how PTHrP is able to modulate β-catenin signaling pathway independently of the Wnt pathway in our CRC cell models. To this end, we analyzed p-Ser552 β-catenin protein levels by Western blot in Caco-2 cells and in HCT116 cells. As shown in **Figure 3A**, incubation of Caco-2 cells with PTHrP for 1 hour (based on conditions previously established) (21), induced phosphorylation of β-catenin at Ser552. The comparison between phospho-Ser552 β-catenin/GAPDH ratio and total β-catenin/GAPDH ratio reveals that the cytokine modulates total β-catenin protein expression but also demonstrates a true increase in phosphorylated β-catenin. To evaluate p-Ser552 β-catenin levels in HCT116 cells, we first performed a time-course trial, as this event had not been previously characterized. The higher metabolic activity of HCT116 cells compared with Caco-2 cells, which we previously demonstrated (18, 20), together with their shorter doubling time (approximately 21 hours vs. 36 hours, respectively), prompted us to examine a broad range of exposure times. As shown in **Figure 3B**, PTHrP increased Ser552 β-catenin at 90 and 120 minutes. No significant effects were observed for longer periods of PTHrP exposure (4 and 8 hours; **Figure 3B**). Regarding total β-catenin protein levels, no significant differences were found in the evaluated times, confirming that the increase in p-Ser552 corresponds to an increase in phosphorylation rather than total protein expression. In view of these results, we then performed immunocytochemistry studies to examine the localization of β-catenin in HCT116 cells. The **Figure 3C** (top panel) shows the images obtained by fluorescence microscopy in untreated and PTHrP-treated HCT116 cells that reveal an increase of β-catenin protein levels in the nuclei of cells exposed to PTHrP for 90 minutes. This observation is consistent with the enhanced phosphorylation of β-catenin at Ser552 detected under the same conditions (**Figure 3B**), suggesting that this phosphorylation site contributes to the cytokine-induced nuclear translocation of β-catenin. In contrast, and according to the results shown in **Figure 3B** and obtained at PTHrP exposure times longer than 2 hours, there are no significant differences between β-catenin protein levels in nuclei of HCT116 control cells and PTHrP-treated HCT116 cells for 3 hours (**Figure 3C**, bottom panel).

Further details about the molecular mechanisms triggered by PTHrP that modulate the activation and translocation of β-catenin in CRC cells are shown as **Supplementary Material**.

We previously demonstrated that PTHrP stimulates the cell cycle progression and proliferation of Caco-2 and HCT116 cells through the activation of several mitogenic pathways such as Phosphatidylinositol 3-kinase (PI3K), Akt (PKB), Extracellular Signal-Regulated Kinases 1 and 2 (ERK1/2 MAPK) and p38 MAPK. Furthermore, we found a complex cross-talk among these pathways when this cytokine acts on CRC cells. The activities of the following three kinases: Protein Kinase C (PKC), Src and PI3K converge in the phosphorylation/activation of Akt leading to the phosphorylation/activation of ERK1/2 MAPK (15, 19–21). These results, along with the data from supplementary material which strongly suggest a relationship between MAPKs and β-catenin signaling pathways, supported the idea that β-catenin might also participate in PTHrP pro-tumor effect on CRC cells. To test this, we performed experiments using iCRT14 (50 μM), the specific inhibitor of β-catenin transcriptional activity, and then Caco-2 cells and HCT116 cells were treated with or without PTHrP 10–^8^ M for 5 days or 1 day, respectively. The dose and time of treatment for each cell line were selected according to our previous research (18, 19, 21, 45). The concentration of iCRT14 was selected according to the literature (46). Trypan blue dye was used to evaluate the number of viable cells because it is unable to enter healthy cells. As shown in **Figure 3D**, the increase in the number of viable cells induced by PTHrP was reversed when the cells were pre-incubated with iCRT14, suggesting that β-catenin transcriptional activity is key for the cell proliferation mediated by PTHrP in both cell lines.

### 3.2 Participation of β-catenin signaling pathway in PTHrP-induced chemoresistance in CRC cells

In our previous reports, we demonstrated that PTHrP favors certain events related with the aggressive behavior of CRC, such as EMT program, tumor-related angiogenesis and chemoresistance through the activation of well-known deregulated pathways in CRC (14, 16, 17, 20). In view of the results shown in **Figure 3** and **Supplementary Material**, we sought to further explore the relation between PTHrP and β-catenin employing CRC cell models, as well as CRC animal models, in order to validate *in vivo* the findings initially obtained *in vitro*. Concerning *in vivo* assays, we have extensive experience using HCT116 cells xenografts in immunocompromised mouse models (nude mice) (14, 16, 17, 20). Although Caco-2 and HCT116 cells are both derived from human CRC, only HCT116 cell line can generate xenografts in nude mice because is more undifferentiated and aggressive. Therefore, in order to properly compare and interpret the results obtained in the nude model, we decided to continue our work exclusively with the HCT116 cell line as human CRC cell model.

Regarding chemoresistance in CRC cells modulated by PTHrP, we previously reported that the cytokine induces resistance to oxaliplatin and other chemotherapeutic drugs through different mitogenic signaling pathways (14, 20). Therefore, given the importance of Wnt/β-catenin in the development of chemoresistance in CRC (24, 25) and since we observed β-catenin nuclear accumulation in CRC cells under PTHrP action, we performed new experiments with the aim to evaluate whether the cytokine via the β-catenin signaling diminishes the sensitivity of CRC cells to drugs usually used in CRC treatment, particularly to platinum-based compounds.

**Figure 4:**
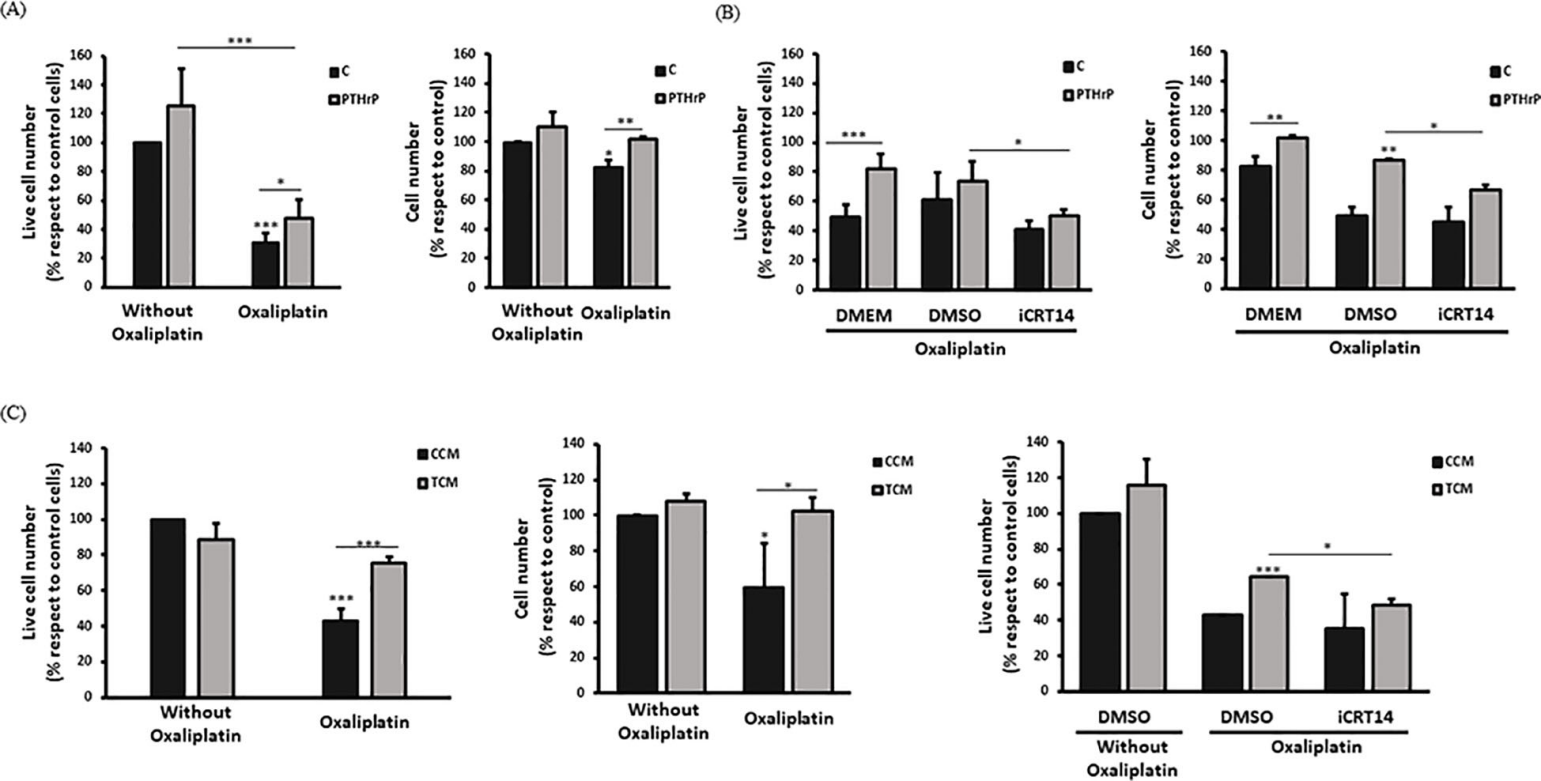
PTHrP attenuates oxaliplatin-induced cytotoxicity through β-catenin signaling. **(A)** HCT116 cells were pretreated with PTHrP (10^-8^ M) or vehicle and subsequently exposed to oxaliplatin (10 µM), and cell viability was evaluated after 24 h. **(B)** HCT116 cells were pre-incubated with iCRT14 (50 μM) for 30 minutes and then treated with PTHrP 10–^8^ M and finally, with oxaliplatin (10 μM) for 24 hours. The number of viable cells was counted by trypan blue staining [left panel **(A, B)**] and neutral red uptake assay [right panel **(A, B)**]. **(C)** HCT116 cells were treated with TCM or CCM, previously obtained from endothelial cells treated or not with PTHrP (10_–8_ M) for 16 hours, respectively. Subsequently, the tumor cells were incubated with 10 µM oxaliplatin in 1% FBS for 24 hours (left and middle panel). HCT116 cells were preincubated with iCRT14, followed by the treatment with TCM or CCM and then oxaliplatin for 24 hours (right panel). DMSO was used as the vehicle of the inhibitor. The number of viable cells was determined by the trypan blue dye exclusion technique (left and right panel) and neutral red uptake assay (middle panel). Data are expressed as mean ± SD. When using the trypan blue dye exclusion technique, statistical significance was determined from two independent biological replicates (n=2), three technical replicates each. For neutral red uptake assay, statistical significance was determined from three independent biological replicates (n=3), five technical replicates each. *p<0.05; **p<0.01; ***p<0.001. CCM, conditioned medium from HMEC-1 without PTHrP collected at 16 hours; TCM, conditioned medium of HMEC-1 with 16 hours of PTHrP treatment; DMSO, dimethylsulfoxide; iCRT14, specific inhibitor of β-catenin transcriptional activity; FBS, fetal bovine serum; PTHrP, parathyroid hormone-related peptide.

Initially, we aimed to reproduce the results obtained with oxaliplatin in HCT116 cells (14) through trypan blue dye exclusion and NRU assays. Hence, HCT116 cells were treated with oxaliplatin (10 µM) with or without PTHrP and it was observed that PTHrP effectively attenuates the cytotoxic effect of the chemotherapeutic agent after 24 hours of treatment (**Figure 4A**). We previously demonstrated that PTHrP exhibits a protective role when CRC cells undergo apoptosis (45); furthermore we observed that the cytokine exerts its action by modulating the intrinsic pathway of apoptosis. As several researchers have demonstrated in HCT116 cells that oxaliplatin induces mitochondrial dysfunction and the apoptotic intrinsic pathway (47), we believe that the increment of live CRC cells by PTHrP even under oxaliplatin effect may be due to both, an upregulation of cell proliferation and a downregulation of the apoptotic intrinsic pathway leading to the resistance of CRC cells to die. In view of these findings, and to evaluate the potential involvement of β-catenin pathway in this chemoresistance, HCT116 cells were incubated with iCRT14 (50 μM), followed by the treatment in presence or absence of PTHrP and finally, exposed to oxaliplatin for 24 hours. As shown in **Figure 4B**, the data obtained by both cell viability assays revealed that PTHrP in DMEM or PTHrP with DMSO (the vehicle of iCRT14) at 24 hours increased the number of HCT116 cells even in the presence of oxaliplatin, confirming again that the cytokine induces resistance to the drug in CRC cells. However, the treatment with iCRT14 significantly decreased the number of viable cells in the presence and absence of PTHrP, restoring oxaliplatin sensitivity in HCT116 cells and suggesting that β-catenin is involved in the PTHrP-induced chemoresistance.

The vascular endothelial cells of the TM release factors involved in tumor progression (48, 49). Considering our background demonstrating the effect of PTHrP on HMEC-1 endothelial cells (16, 17), we then proposed to evaluate whether the factors from these TM cells exposed to PTHrP favor oxaliplatin chemoresistance of CRC cells. To this end, HCT116 cells were exposed to the CM derived from endothelial cells, previously treated with PTHrP, for 16 hours (TCM). Following TCM treatment, HCT116 cells were exposed to oxaliplatin (10 µM) in 1% FBS until completing 24 hours of treatment. Treatment times were selected based on our previous results (16, 17, 20). The results obtained by trypan blue dye exclusion test and NRU assay revealed that HCT116 cells are less sensitive to oxaliplatin when they are exposed to the TCM (**Figure 4C**, left and middle panel). These findings suggest that the cytokine, acting through TM cells, would favor the CRC aggressive behavior by attenuating oxaliplatin cytotoxic effect on HCT116 tumor cells. In view of these results, we proceeded to analyze whether β-catenin pathway is involved in the effect of TCM on the reduced sensitivity to oxaliplatin of CRC cells. To that end, HCT116 cells were preincubated with iCRT14, followed by the treatment with TCM or CCM and then oxaliplatin for 24 hours. By trypan blue dye we observed that the number of HCT116 cells exposed to TCM and DMSO is greater than the number of HCT116 cells exposed to CCM and DMSO, even in the presence of the cytostatic agent. However, this effect was reversed in the presence of iCRT14 (**Figure 4C**, right panel). These observations suggest that growth factors and/or cytokines present in the CM derived from PTHrP-treated endothelial cells attenuate the cytotoxic effect of oxaliplatin on HCT116 cells, through the β-catenin signaling.

### 3.3 Study of PTHrP effect in a nude mice model under oxaliplatin treatment

**Figure 5:**
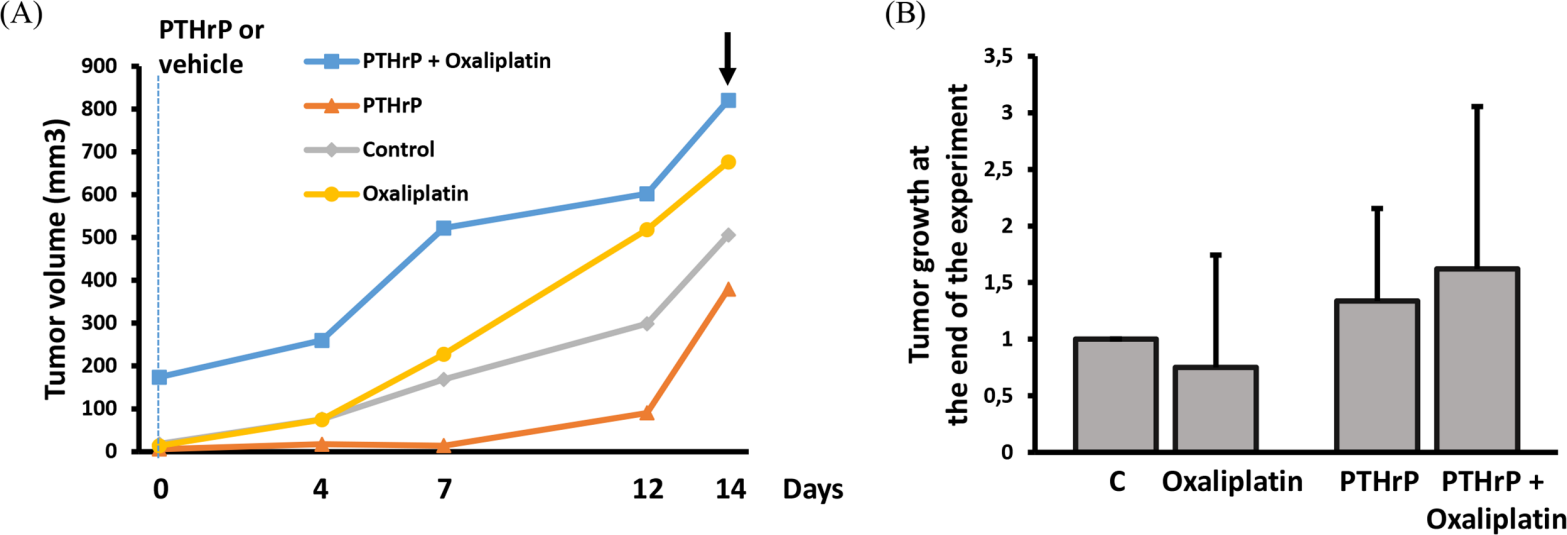
Study of the effects of PTHrP and oxaliplatin in HCT116 cells tumor xenografts in immunodeficient mice. HCT116 cells were inoculated subcutaneously into the right flank of nude mice; once the tumors reached the appropriate size, daily intratumoral injections of PTHrP were initiated. The chemotherapy drug was administered once a week. Tumor growth was monitored by measuring tumor volume. **(A)** tumor volume over time is shown from the start of PTHrP or vehicle administration. No significant differences were observed among the experimental groups. **(B)** tumor growth rate was calculated at the end of the experiment. Growth rate results are represented in bar graphs. The black arrows show the effect of PTHrP and oxaliplatin. C, vehicle of PTHrP; PTHrP, parathyroid hormone-related peptide.

As we mentioned, studies using HCT116 cell xenografts in “nude” immunocompromised mice provided evidence that PTHrP modulates the expression of multiple proteins associated with chemoresistance (14–18, 20). Therefore, it was proposed to use this same CRC animal model to validate the oxaliplatin resistance induced by PTHrP observed *in vitro*. As we described in the Material and Methods Section, we established a model of subcutaneous xenografts by inoculating HCT116 cells into the right flank of nude mice. Once the tumors were palpable, mice were treated with oxaliplatin, or with PTHrP (alone or in combination with oxaliplatin) or with PBS for 2 weeks (**Figure 1**). During the experimental frame, tumor volumes were measured, and when we compared tumor size between the different groups, no significant differences were observed (**Figure 5A**). Similar results were obtained when we analyzed the tumor growth rate at the end of the protocol between the four groups (**Figure 5B**). However, in the group that received PTHrP and the cytostatic agent, we noted a tendency of PTHrP to promote tumor growth even in the presence of oxaliplatin with respect to the group that only received the drug (**Figure 5A**, black arrow). Given that tumor treatment with PTHrP was administered for 14 days, further experiments are needed to determine prolonged effects of the cytokine regarding oxaliplatin treatment.

### 3.4 PTHrP decreases the oxaliplatin sensitivity of highly aggressive murine CT-26 cells

**Figure 6:**
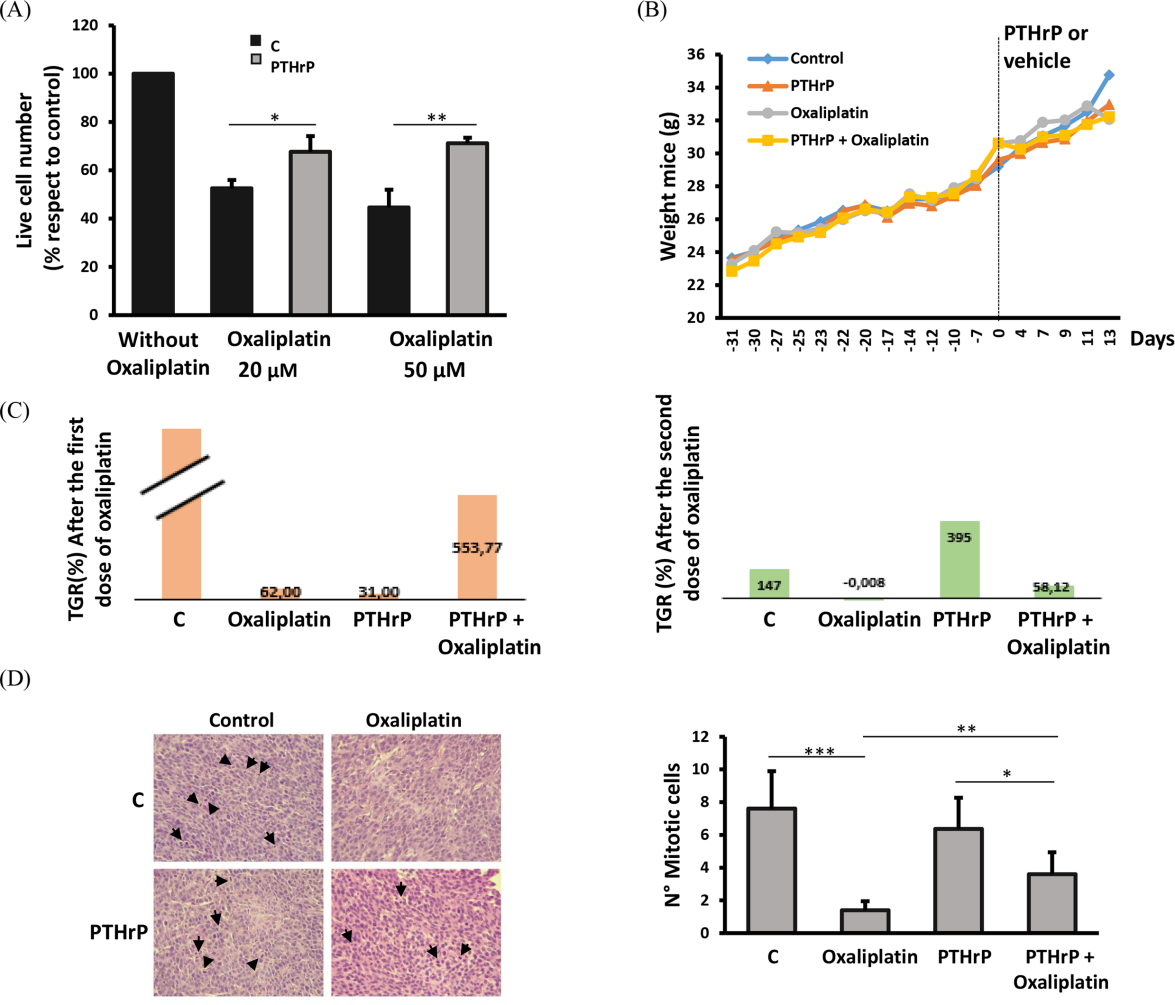
PTHrP increases the number of mitotic CT-26 cells and promotes tumor growth in Balb/c mice despite oxaliplatin treatment. **(A)** CT-26 cells were treated or not with PTHrP (10-8 M) and subsequently exposed to increasing concentrations of oxaliplatin for a total of 24 hours. Cell viability was assessed by trypan blue exclusion assay. Data are expressed as mean ± SD. Statistical significance was determined from three independent biological replicates (n=3), 3 technical replicates each. **(B)** CT-26 cells were subcutaneously inoculated into Balb/c mice to generate tumors. Once tumors reached the appropriate size, intratumoral treatment with PTHrP or its vehicle was initiated. Finally, intraperitoneal cytostatic agent or PBS was administered once a week. As a parameter of animal welfare, the mice were weighed three times a week. **(C)** Tumors were measured every three days, and tumor volume values were used to determine the tumor growth rate (TGR) after the first and second doses of oxaliplatin. **(D)** Representative hematoxylin and eosin-stained tumor sections. Mitotic events are indicated by arrows. Data are expressed as mean ± SD. Statistical significance was determined from the analysis of ten random microscopic fields per section, from three independent tumor sections per animal. *p<0.05; **p<0.01; ***p<0.001. C, vehicle of PTHrP; Control, without oxaliplatin; PTHrP, parathyroid hormone-related peptide; PBS, phosphate-buffered saline solution.

The fact that our research group has extensive information about the effects of PTHrP in HCT116 cells both *in vitro* and *in vivo*, motivated us to consider the need to employ another more aggressive CRC cell model but with metastatic capacity to further study the effects of PTHrP on events associated with invasion and metastasis. For this reason, we chose the CT-26 cell line derived from the murine CRC with metastatic capacity (34, 50). CT-26 cells were treated with or without PTHrP and then were exposed to the oxaliplatin (20 and 50 µM) for 24 hours. Control cells were exposed to oxaliplatin vehicle for the same time. As shown in **Figure 6A**, using trypan blue dye exclusion technique we observed, for the first time, that these cells are responsive to PTHrP. Furthermore, CT-26 cells in the presence of PTHrP are less sensitive to oxaliplatin at doses of 20 µM and 50 µM compared to the condition with oxaliplatin alone (p< 0.01) (**Figure 6A**). These data suggest that in CT-26 cells, PTHrP may promote events associated with aggressive behavior, such as oxaliplatin chemoresistance.

### 3.5 Study of PTHrP effect in a metastatic CRC model in mice under oxaliplatin treatment

The CRC animal model of HCT116 cells xenografts in athymic mice has limitations to evaluate the interactions between the tumor and the stroma, because it involves human xenografts in mice. Considering our *in vitro* results obtained with CT-26 cells (**Figure 6A**), we then decided to use a new syngeneic and immunogenic CRC model based on the inoculation of these cells into Balb/c mice (51). This model allows us to evaluate whether PTHrP has effects on oxaliplatin resistance considering the impact on the TM. Tumors were generated as described in Materials and Methods and in **Figure 2**. The weight of the mice was determined three times a week to assess their well-being, and no statistically significant differences in weight were observed during the treatment. However, in the second group receiving intraperitoneal oxaliplatin a decrease in weight was observed after the second dose, leading to the discontinuation of the experiment (day 14 after the start of PTHrP or vehicle administration) (**Figure 6B**). In addition, tumors were measured and tumor volumes and the TGR (%) were calculated, observing that oxaliplatin alone clearly reduced tumor growth (**Figure 6C**). Further, after the second dose of the drug, we found that the TGR was higher in the third group (PTHrP-treated mice) compared to the first group (control mice) and in the fourth group (mice treated with PTHrP and oxaliplatin) compared to the second group (mice treated with oxaliplatin) (**Figure 6C**). These *in vivo* findings suggest that PTHrP may be associated with increased tumor growth and reduced oxaliplatin efficacy in an immunocompetent CRC model. Further investigations to establish causality and underlying mechanisms are needed.

At the start of treatment, two mice showed signs of abdominal ascites and they were humanely euthanized. During the necropsy of these mice, small tumor masses were observed in the abdominal cavity in addition to the primary tumor. Furthermore, considering that CT-26 cells have metastatic potential in the liver, lungs and spleen (34, 52), it was decided to evaluate these organs. Upon examination of the liver and lungs, no macroscopic evidence of nodules was found on their surfaces (data not shown). However, a more thorough examination of the spleen revealed that mice that developed tumors on the right flank had a larger spleen compared to mice that did not develop tumors. Additional experiments are needed to determine whether PTHrP and oxaliplatin treatments influence these differences.

Next, the H&E-stained sections of the tumor tissues were observed (**Figure 6D**). Multiple areas of the tumors analyzed displayed cells in the growth phase, cells with apoptotic features, and regions with necrosis. Areas with increased cell death corresponded to the tumor core, consistent with nutrient deprivation; whereas the tumor peripheries in both control and PTHrP-treated groups showed abundant mitotic figures (**Figure 6D**, black arrow), indicating high proliferation, with no significant differences between groups. On the other hand, the greater number of mitotic figures in mice exposed to oxaliplatin and PTHrP (fourth group) compared to mice exposed to oxaliplatin alone suggests that the cytokine promotes cell division and tumor growth even in the presence of the cytostatic agent (p<0.01) (**Figure 6D**).

### 3.6 PTHrP modulates β-catenin protein levels in CRC animal models

**Figure 7:**
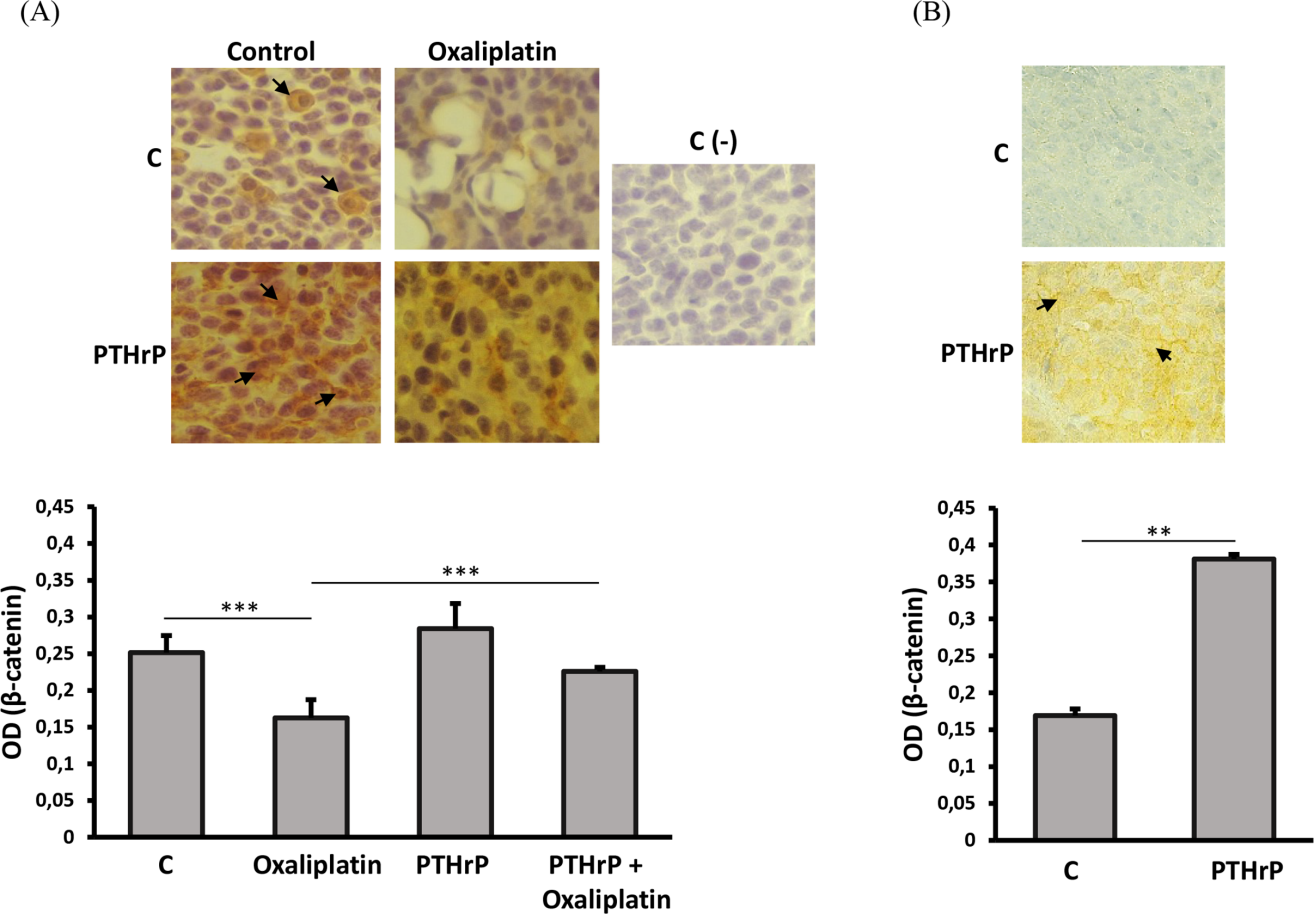
The β-catenin protein levels analysis *in vivo*. **(A)** representative images (600x) and quantification of β-catenin immunohistochemical staining in tumor tissues derived from CT-26 cells in Balb/c mice. **(B)** representative images (400×) and quantification of β-catenin immunohistochemical staining in HCT116 xenografts generated in immunodeficient nude mice. The β-catenin subcellular localization is indicated by black arrows. Data are expressed as mean ± SD. Statistical significance was determined from the analysis of ten random fields per section, from three independent tumor sections per animal. *p< 0.05; **p< 0.01; ***p< 0.001. C, vehicle of PTHrP; C (-), negative control; PTHrP, parathyroid hormone-related peptide; Control, without oxaliplatin.

Considering that β-catenin is a pro-tumor factor in CRC (53, 24) and based on our findings showed in **Figure 6**, we next examined β-catenin protein levels by IHC in tumor samples analyzed in the previous Section. As we expected, β-catenin immunoreactivity quantification in tumors from mice which received the cytotoxic drug (second group) is lower than in tumors from control mice (first group). However, we observed that in tumors from mice treated with oxaliplatin and PTHrP, β-catenin protein levels are higher than in tumors from oxaliplatin-treated mice (fourth versus second group) (p<0.001). The protein localization was predominantly cytosolic and is indicated by black arrows in the images (**Figure 7A**). In this syngeneic model, the observed findings are consistent with a potential association between PTHrP action and altered β-catenin signaling. Further investigation is warranted to validate and characterize this interaction.

Finally, considering the effect of PTHrP in the β-catenin signaling pathway observed in HCT116 cells (see **Figure 3**, **Supplementary Material**), we evaluated β-catenin expression by IHC in tumors from HCT116 xenografts in nude mice. Although this analysis did not provide definitive conclusions regarding PTHrP effects under oxaliplatin scheme (**Figure 5**), β-catenin levels were significantly increased in tumors from PTHrP-treated mice compared with controls (third vs. first group; p<0.01). The protein localization was predominantly cytosolic and is indicated by black arrows in the images (**Figure 7B**).

Taken together, these results suggest that PTHrP may modulate β-catenin expression *in vivo*; however, more investigations are needed to confirm pathway activation or to demonstrate a direct causal role for β-catenin in chemoresistance.

## 4 Discussion

Oxaliplatin-based chemotherapy is the standard treatment regimen for CRC patients with advanced disease (54). However, drug resistance remains a major challenge in CRC treatment, arising from multiple mechanisms such as altered drug transport, target sensitivity changes, enhanced DNA repair, mitochondrial interactions, signaling pathway deregulation, and TM influences. Despite progress in understanding these processes, effective clinical strategies to overcome oxaliplatin resistance are still limited (25, 55, 56).

Wnt signaling is divided into two pathways, the canonical one, which participates in β-catenin/TCF target gene activation and the non-canonical pathway that induces signaling independent of β-catenin (57). In addition, there are genetic mutations and alternative signaling pathways known to induce Wnt-independent activation of β-catenin, which are highly relevant to the development and progression of various types of cancer (58, 59). Most literature concludes β-catenin signaling pathway as a central mediator of chemoresistance in CRC (25, 60–62). Our research group demonstrated that PTHrP acts as a pro-tumor cytokine in human CRC-derived cells and promotes β-catenin nuclear localization independently of Wnt signaling (14–21). Furthermore, PTHrP activates several kinases in these CRC cell lines (15, 19–21), which may activate β-catenin through phosphorylation at Ser552. This mechanism is known to promote β-catenin dissociation from cell–cell junctions, facilitating its nuclear translocation and β-catenin/TCF-mediated transcription (59, 63, 64). In view of this background, we analyzed the protein status of p-Ser552 β-catenin both in Caco-2 cells and HCT116 cells under PTHrP action. We observed that phosphorylated Ser552 β-catenin expression profile increased by PTHrP treatment in both CRC cell lines; in addition, the pathways of Akt, ERK1/2 MAPK and p38 MAPK are involved in this process. We also confirmed that the cytokine action resulted in the accumulation of p-Ser552 β-catenin in CRC cell nuclei. The significance of this phosphorylation in cancer was described by other works; in prostate cancer, the phosphorylation of Ser 552 β-catenin can facilitate the activation of ERK1/2 and Stat3, promoting cell survival and proliferation (63). Furthermore, intestinal polyposis and inflammation-induced dysplastic transformation in the colon have been linked to β-catenin phosphorylation at these residues (59). Based on these findings, we then evaluate the number of viable CRC cells after PTHrP in the presence or absence of a specific inhibitor of β-catenin activity. iCRT14 is known to act at the nuclear level by inhibiting direct interactions between β-catenin and Transcription Factor-4 (TCF4, a transcription factor implicated in Wnt signaling), antagonizing the transcriptional function of nuclear β-catenin and, consequently, blocking the signaling pathway (65). Since it has been previously reported β-catenin participation as a mediator of tumor cells proliferation (53, 66), our research revealed that β-catenin transcriptional activity is fundamental for the proliferation of Caco-2 and HCT116 cells induced by PTHrP.

Several studies reported that β-catenin is involved in the decrease of oxaliplatin sensitivity in tumor intestinal cells (25, 60, 62, 67). In line with this, we previously reported that PTHrP induces resistance to oxaliplatin and other chemotherapeutic drugs through well-known deregulated pathways in CRC (14, 20). In the present study, the relationship between PTHrP and β-catenin was established through two different cell viability assays, where β-catenin pathway mediates sensitivity changes to oxaliplatin promoted by the cytokine in CRC cells.

The TM is composed of tumor cells, interstitial cells, extracellular matrix and factors released by all these components which mediated the tumor progression; thus, TM plays a crucial role in tumor proliferation, invasion, and metastasis (68). In previous studies, we observed that human endothelial HMEC-1 cells from microvasculature express the PTH receptor type 1 (PTHR1), a G protein coupled receptor (GPCR) which it is shared by PTH and PTHrP (17); although the intrinsic production of PTHrP in this specific cell line is still unknown, its synthesis and release have been recorded in other human endothelial cells (69). Based on PTHR1 presence in HMEC-1 endothelial cells, our group focused on exploring PTHrP role in the modulation of the CRC cells aggressive phenotype through its influence on these TM cells and we demonstrated that biomolecules present in the endothelial cells-derived CM promote, in CRC cells, characteristics associated with cellular plasticity, dedifferentiation and resistance to CPT-11 drug (15–17). Therefore, the next step was to investigate whether released factors from endothelial cells exposed to PTHrP contribute to the oxaliplatin resistance of HCT116 cells. The attenuation of oxaliplatin cytostatic effect on HCT116 cells by TCM and observed by us, suggests the presence of cytokines and/or factors in this TCM that promote changes associated with the chemoresistance phenomenon. According with our hypothesis, it is now well established that the communication between tumor cells and stromal components, including endothelial cells, by the secretion of factors into the tumor niche (cytokines, growth factors, extracellular vesicles) favors drug resistance across cancer types, and that endothelial cells-derived factors such as Vascular Endothelial Growth Factor (VEGF) have a key role in promoting tumor cell survival and anti-apoptotic events under chemotherapeutic stress (70).

In this work we also discovered that the increase in the number of viable HCT116 cells when exposed to TCM, even in the presence of oxaliplatin, is reversed in the presence of iCRT14, suggesting that β-catenin signaling is involved in oxaliplatin resistance in CRC cells. We observed that in CRC cells, PTHrP promotes the expression and activation of Met, a tyrosine kinase receptor that is overexpressed or aberrantly activated in CRC and is associated with its progression and chemoresistance (14, 71). Met can be activated by its ligand, the Hepatocyte Growth Factor (HGF), but can also be transactivated by PTHrP (14). Moreover, the Met signaling pathway is related to β-catenin activation in tumor cells (72, 73). Mezquita and collaborators observed that oxaliplatin-resistant CRC cells overexpress Met and VEGF Receptor (67). Considering our findings shown herein and the mentioned background, we hypothesize that VEGF, HGF and/or PTHrP could be present in the CM, promoting resistance to oxaliplatin through β-catenin activation. However, further studies are needed to confirm this and elucidate the secretion profile of HMEC-1 cells exposed to PTHrP. Therefore, understanding the relationship between cancer cells and TM components is fundamental for designing strategies for CRC therapy.

In recent years, we have demonstrated that PTHrP in HCT116 cells xenografts in nude mice modulates molecular and morphological events directly related to chemoresistance (14, 16–18, 20). Lee and colleagues, observed in a mouse xenograft model of prostate cancer that PTHrP mediated enzalutamide (androgen receptor antagonist) resistance in tissue culture and TCF4 overexpression, resulted in enzalutamide resistance via a Wnt-independent pathway (74). Meanwhile, other researchers demonstrated that the use of the drug valproic acid and the standard oxaliplatin/fluoropyrimidine chemotherapy regimen both *in vitro* and *in vivo*, leads to both, a marked reduction in β-catenin levels and the subsequent less activation of β-catenin/TCF/LEF target promoters, contributing to improve the effectiveness of drugs commonly used in clinical practice for CRC treatment (25). Therefore, using HCT116 cells xenografts in nude mice, we set out to extrapolate the oxaliplatin resistance results obtained *in vitro* and to elucidate whether PTHrP administration together with the secretions of factors from TM or tumor cells participate in the modulation of β-catenin pathway. However, during the experiment, we were unable to observe the PTHrP effect on cytostatic resistance. This may be due to the limitations of the “nude” mouse model used, which lacks both, mature T cells in the thymus and a T cell-dependent B cell response but has an intact innate immune response that conditions the appropriate development of a tumor niche from human cancer cells (75). Another potential reason for the observed differences between *in vitro* and *in vivo* results may be that in our *in vitro* assays, PTHrP was added directly to the culture medium, providing a homogeneous and sustained dose throughout the cell culture. In contrast, in the HCT116 xenograft model PTHrP was administered by daily intratumoral injections. In solid tumors, intratumoral delivery can create steep concentration gradients and heterogeneous exposure because the drug movement is limited by interstitial transport barriers and tumor structure, and because the injected compound can be removed by local perfusion and lymphatic and vascular drainage. These processes shorten intratumoral residence time after injection (76, 77). Under these conditions, PTHrP was associated with altered β-catenin immunoreactivity in tumor tissue, although this did not translate into measurable differences in tumor growth during the experimental period (78). Additional studies with extended treatment schedules will be necessary to determine whether PTHrP modulates oxaliplatin sensitivity in this model and to define the relevance of β-catenin signaling in such effects.

Given these findings, the use of a syngeneic and immunocompetent CT-26 murine model allowed partial assessment of tumor–microenvironment interactions that cannot be fully captured in xenograft systems. Within this context, oxaliplatin therapy consistently reduced tumor growth, supporting the responsiveness of this model to the treatment. Notably, PTHrP-treated groups showed higher TGR compared to their respective controls, both in the presence and absence of oxaliplatin. However, these findings should be interpreted with caution. The early termination of the experiment due to treatment-associated toxicity prevents a comprehensive evaluation of long-term tumor dynamics. Moreover, while the observed increase in TGR and β-catenin immunoreactivity in PTHrP-treated animals suggests a potential association with reduced oxaliplatin efficacy, this effect requires further validation.

The results observed between the nude mouse xenograft model and the syngeneic Balb/c model underscore the importance of the host microenvironment, including the immune context, in the modulation of chemoresistance (79). While PTHrP did not significantly alter tumor volume in the immunodeficient xenograft model, the syngeneic model with functional immunity showed a higher TGR and reduced response to oxaliplatin in animals treated with PTHrP. These results are consistent with previous evidence demonstrating that the presence of stromal and immune components significantly influences the therapeutic response. Immune and stromal components can communicate with tumor cells through cytokines, chemokines, and growth factors, reinforcing survival pathways and cytotoxicity evasion (80).

Although we were unable to observe metastatic nodules in the organs evaluated from the necropsy of the Balb/c mice, we observed an increase in spleen size in those mice where the tumor grew. Alimohammadi and colleagues demonstrated that oxaliplatin monotherapy causes an increase in the population of regulatory T lymphocytes in the spleen of mice (27). Furthermore, Funk and colleagues demonstrated that PTHrP increases its levels in the spleen during an exacerbated immune response. This cytokine could play an important role in immune modulation, perhaps by mediating changes in lymphocyte proliferation and/or function (81).

In conclusion, our *in vitro* studies expand the knowledge regarding the molecular mechanisms triggered by PTHrP in CRC cells, associated with β-catenin stabilization and increased nuclear localization that results in its Wnt-independent activation; also, the findings support a link between PTHrP exposure and reduced oxaliplatin sensitivity, both through direct action on tumor cells and via tumor microenvironment-derived endothelial cells. While pharmacological inhibition suggests the involvement of β-catenin signaling in this process. *In vivo*, the effects of PTHrP on oxaliplatin response were model-dependent. Although the syngeneic model suggests a potential association between PTHrP and reduced oxaliplatin efficacy, further studies are needed to clarify the roles of PTHrP and β-catenin in this effect, regarding the mechanism or causality. The observation of different responses between immunodeficient mouse model and immunocompetent syngeneic model of CRC highlight the importance of cell behavior and chemotherapy response in a context-dependent manner.

## Data Availability

The original contributions presented in the study are included in the article/**Supplementary Material**. Further inquiries can be directed to the corresponding author.

## Appendix

The phosphorylation of β-catenin at specific residues induced by PTHrP in human CRC-derived cells, shown in **Figure 3**, reveals how the cytokine regulates at least one known mechanism for stabilizing β-catenin and its subsequent nuclear localization. To investigate whether the kinases Akt, p38 MAPK and ERK1/2 MAPK, which are activated by the cytokine (15, 19–21) participate in this particular phosphorylation, CRC cells were pre-incubated with GSK690693 (50 nM, Akt inhibitor), PD98059 (20 mM, ERK 1/2 MAPK inhibitor) or SB203580 (20 μM, p38 MAPK inhibitor) and then treated with PTHrP 10_–8_ M. Western blot assays using anti-p-β-catenin (Ser552) antibody show the involvement of Akt, ERK1/2 MAPK (**Figure A**) and p38 MAPK (**Figure B**) pathways in the phosphorylation of Ser552 sites induced by PTHrP in CRC cells. The analysis of total β-catenin/GAPDH ratio validates the changes in p-Ser 552 β-catenin observed between the different experimental conditions. Furthermore, the subcellular fractionation followed by Western blot analysis using the following antibodies: anti-p-Ser 552 β-catenin, anti- β-catenin and anti- Lamin B (as a control of nuclear fraction purity) and the corresponding quantification of the blots reveal that the cytokine-triggered signaling pathways lead to the accumulation of β-catenin and its phosphorylated form in the nucleus and this effect was mainly observed before 3 hours of exposure (**Figure C**), according with PTHrP exposition times where the changes in p-Ser552 were statistically significant (**Figure 3**) (43, 44, 82). The data presented in **Figures A**–**C** expand the knowledge regarding the molecular mechanisms triggered by PTHrP in CRC cells that lead to the stabilization and subsequent nuclear localization of β-catenin and highlight the relevance of the cytokine in β-catenin activation independently of Wnt.

## References

[1] Sung H Ferlay J Siegel R Laversanne M Soerjomataram I Jemal A . Global cancer statistics 2020: GLOBOCAN estimates of incidence and mortality worldwide for 36 cancers in 185 countries. CA Cancer J Clin. (2021) 71:209–49. doi: 10.3322/caac.21660. PMID: 33538338

[2] International Agency for Research on Cancer . Available online at: https://www.iarc.who.int/news-events/global-burden-ofcolorectal-cancer-in-2020-and-2040-incidence-and-mortality-estimates-from-globocan/ (Accessed July 10, 2025).

[3] Briede I Balodis D Gardovskis J Strumfa I . Stemness, inflammation and epithelial-mesenchymal transition in colorectal carcinoma: The intricate network. Int J Mol Sci. (2021) 22:12891. doi: 10.3390/ijms222312891. PMID: 34884696 PMC8658015

[4] Ma SC Zhang JQ Yan TH Miao MX Cao YM Cao YB . Novel strategies to reverse chemoresistance in colorectal cancer. Cancer Med. (2023) 12:11073–96. doi: 10.1002/cam4.5594. PMID: 36645225 PMC10242875

[5] Zeng K Li W Wang Y Zhang Z Zhang L Zhang W . Inhibition of CDK1 overcomes oxaliplatin resistance by regulating ACSL4-mediated ferroptosis in colorectal cancer. Adv Sci (Weinh). (2023) 10:e2301088. doi: 10.1002/advs.202301088. PMID: 37428466 PMC10477855

[6] Yang Y Yuan H Zhao L Guo S Hu S Tian M . Targeting the miR-34a/LRPPRC/MDR1 axis collapse the chemoresistance in P53 inactive colorectal cancer. Cell Death Diff. (2022) 29:2177–89. doi: 10.1038/s41418-022-01007-x. PMID: 35484333 PMC9613927

[7] Zhong X He X Wang Y Hu Z Huang H Zhao S . Warburg effect in colorectal cancer: the emerging roles in tumor microenvironment and therapeutic implications. J Hematol Oncol. (2022) 15(1):160. doi: 10.1186/s13045-022-01358-5 36319992 PMC9628128

[8] Laplagne C Domagala M Le Naour A Quemerais C Hamel D Fournié JJ . Latest advances in targeting the tumor microenvironment for tumor suppression. Int J Mol Sci. (2019) 20:4719. doi: 10.3390/ijms20194719. PMID: 31547627 PMC6801830

[9] Naafs MAB . Parathyroid Hormone Related Peptide (PTHrP): A Mini-Review. Endocrinol Metab Int J. (2017) 5(6). doi: 10.15406/emij.2017.05.00139

[10] McCauley LK Martin TJ . Twenty-five years of PTHrP progress: from cancer hormone to multifunctional cytokine. J Bone Miner. Res. (2012) 27:1231–9. doi: 10.1002/jbmr.1617. PMID: 22549910 PMC4871126

[11] Zhang R Li J Assaker G Camirand A Sabri S Karaplis A . Parathyroid hormone-related protein (PTHrP): an emerging target in cancer progression and metastasis. Adv Exp Med Biol. (2019) 1164:161–78. doi: 10.1007/978-3-030-22254-3_13. PMID: 31576548

[12] Nishihara M Kanematsu T Taguchi T Razzaque MS . PTHrP and tumorigenesis: is there a role in prognosis? Ann N. Y. Acad Sci. (2007) 1117:385–92. doi: 10.1196/annals.1402.046. PMID: 18056053

[13] Nishihara M Ito M Tomioka T Ohtsuru A Taguchi T Kanematsu T . Clinicopathological implications of parathyroid hormone-related protein in human colorectal tumours. J Pathol. (1999) 187:217–22. doi: 10.1002/(SICI)1096-9896(199901)187:2<217::AID-PATH210>3.0.CO;2-0 10365097

[14] Novoa Díaz MB Carriere P Gigola G Zwenger A Calvo N Gentili C . Involvement of Met receptor pathway in aggressive behavior of colorectal cancer cells induced by parathyroid hormone-related peptide. World J Gastroenterol. (2022) 28:3177–200. doi: 10.3748/wjg.v28.i26.3177. PMID: 36051345 PMC9331538

[15] Novoa Díaz MB Carriere PM Martín MJ Calvo N Gentili C . Involvement of parathyroid hormone-related peptide in the aggressive phenotype of colorectal cancer cells. World J Gastroenterol. (2021) 27(41):7025–40. doi: 10.3748/wjg.v27.i41.7025 PMC8613645 34887626

[16] Carriere P Calvo N Novoa Díaz MB Lopez-Moncada F Herrera A Torres MJ . Role of SPARC in the epithelial-mesenchymal transition induced by PTHrP in human colon cancer cells. Mol Cell Endocrinol. (2021) 530:111253. doi: 10.1016/j.mce.2021.111253. PMID: 33781836

[17] Calvo N Carriere P Martín MJ Gigola G Gentili C . PTHrP treatment of colon cancer cells promotes tumor associated-angiogenesis by the effect of VEGF. Mol Cell Endocrinol. (2019) 483:50–63. doi: 10.1016/j.mce.2019.01.005. PMID: 30639585

[18] Calvo N Carriere P Martin MJ Gentili C . RSK activation via ERK modulates human colon cancer cells response to PTHrP. J Mol Endocrinol. (2017) 59:13–27. doi: 10.1530/JME-16-0216. PMID: 28385776

[19] Calvo N Martín MJ Russo de Boland A Gentili C . Involvement of ERK1/2, P38 MAPK, and PI3K/Akt signaling pathways in the regulation of cell cycle progression by PTHrP in colon adenocarcinoma cells. Biochem Cell Biol. (2014) 92:305–15. doi: 10.1139/bcb-2013-0106. PMID: 25051885

[20] Martín M Gigola G Zwenger A Carriquiriborde M Gentil F Gentili C . Potential therapeutic targets for growth arrest of colorectal cancer cells exposed to PTHrP. Moll. Cell Endocrinol. (2018) 478:32–44. doi: 10.1016/j.mce.2018.07.005. PMID: 30009852

[21] Martín MJ Calvo N de Boland AR Gentili C . Molecular mechanisms associated with PTHrP-induced proliferation of colon cancer cells. J Cell Biochem. (2014) 115:2133–45. doi: 10.1002/jcb.24890. PMID: 25053227

[22] Hasbal-Celikok G Aksoy-Sagirli P Altiparmak-Ulbegi G y Can A . Identification of AKT1/β-catenin mutations conferring cetuximab and chemotherapeutic drug resistance in colorectal cancer treatment. Oncol Lett. (2021) 21:209. doi: 10.3892/ol.2021.12470. PMID: 33574948 PMC7816326

[23] Ambrosi G Voloshanenko O Eckert AF Kranz D Nienhaus GU Boutros M . Allele-specific endogenous tagging and quantitative analysis of β-catenin in colorectal cancer cells. eLife. (2022) 11:e64498. doi: 10.7554/eLife.64498. PMID: 35014953 PMC8752093

[24] Xu J Lv G Xu B Jiang B . Overexpression of UBE2M through Wnt/β-catenin signaling is associated with poor prognosis and chemotherapy resistance in colorectal cancer. Transl Cancer Res. (2020) 9:5614–25. doi: 10.21037/tcr-20-2641. PMID: 35117925 PMC8797438

[25] Roca MS Lombardi R Testa C Iannelli F Grumetti L Moccia T . Valproic acid improves the efficacy of oxaliplatin/fluoropyrimidine-based chemotherapy by targeting cancer stem cell via β-catenin modulation in colorectal cancer. Cell Death Dis. (2025) 16:583. doi: 10.1038/s41419-025-07902-8. PMID: 40750758 PMC12316941

[26] Uzun S Isik A Katipoglu K Guner G Akyol A . Characterization of the subcellular distribution of phospho-β-catenin in colorectal cancer. In. Vivo. (2023) 37:1576–83. doi: 10.21873/invivo.13242. PMID: 37369481 PMC10347937

[27] Alimohammadi R Mahmoodi Chalbatani G Alimohammadi M Ghaffari-Nazari H Rahimi A Mortaz E . Dual blockage of both PD-L1 and CD47 enhances the therapeutic effect of oxaliplatin and FOLFOX in CT-26 mice tumor model. Sci Rep. (2023) 13:2472. doi: 10.1038/s41598-023-29363-9. PMID: 36774400 PMC9922272

[28] Bradford M . A rapid and sensitive method for quantification of microgram quantities of proteins utilizing the principle of protein binding. Anal Biochem. (1976) 72:248–54. doi: 10.1016/0003-2697(76)90527-3. PMID: 942051

[29] Laemmli UK . Cleavage of structural proteins during the assembly of the head of bacteriophage T4. Nature. (1970) 227(5259):680–5. doi: 10.1038/227680a0 5432063

[30] Fitzpatrick M . The open lab book (2021). Available online at: https://theolb.readthedocs.io/en/latest/ (Accessed July 10, 2025).

[31] Dunn EF Iida M Myers RA Campbell DA Hintz KA Armstrong EA . Dasatinib sensitizes KRAS mutant colorectal tumors to cetuximab. Oncogene. (2011) 30:561–74. doi: 10.1038/onc.2010.430. PMID: 20956938 PMC3025039

[32] Erdem Z Schwarz S Drev D Heinzle C Reti A Heffeter P . Irinotecan upregulates fibroblast growth factor receptor 3 expression in colorectal cancer cells, which mitigates irinotecan-induced apoptosis. Transl Oncol. (2017) 10:332–9. doi: 10.1016/j.tranon.2017.02.004. PMID: 28340475 PMC5367848

[33] Nagaraju G Alese O Landry J Diaz R El-Rayes B . HSP90 inhibition downregulates thymidylate synthase and sensitizes colorectal cancer cell lines to the effect of 5FU-based chemotherapy. Oncotarget. (2014) 5:9980–91. doi: 10.18632/oncotarget.2484. PMID: 25296971 PMC4259452

[34] Ripoll GV Garona J Hermo GA Gomez DE Alonso DF . Effects of the synthetic vasopressin analog desmopressin in a mouse model of colon cancer. Anticancer Res. (2010) 30(12):5049–54. 21187489

[35] de Carvalho T Lara P Jorquera-Cordero C Aragão C de Santana Oliveira A Garcia V . Inhibition of murine colorectal cancer metastasis by targeting M2-TAM through STAT3/NF-kB/AKT signaling using macrophage 1-derived extracellular vesicles loaded with oxaliplatin, retinoic acid, and Libidibia ferrea. BioMed Pharmacother. (2023) 168:115663. doi: 10.1016/j.biopha.2023.115663. PMID: 37832408

[36] Lamarca A Ronot M Moalla S Crona J Opalinska M Lopez Lopez C . Tumor growth rate as a validated early radiological biomarker able to reflect treatment-induced changes in neuroendocrine tumors: The GREPONET-2 study. Clin Cancer Res. (2019) 25:6692–9. doi: 10.1158/1078-0432.CCR-19-0963. PMID: 31375514

[37] Amable G Martínez-León E Picco ME Di Siervi N Davio C Rozengurt E . Metformin inhibits β-catenin phosphorylation on Ser-552 through an AMPK/PI3K/Akt pathway in colorectal cancer cells. Int J Biochem Cell Biol. (2019) 112:88–94. doi: 10.1016/j.biocel.2019.05.004. PMID: 31082618

[38] Tauriello DV Calon A Lonardo E Batlle E . Determinants of metastatic competency in colorectal cancer. Mol Oncol. (2017) 11:97–119. doi: 10.1002/1878-0261.12018. PMID: 28085225 PMC5423222

[39] Yan X Zhao J Zhang R . Interleukin-37 mediates the antitumor activity in colon cancer through β-catenin suppression. Oncotarget. (2017) 8:49064–75. doi: 10.18632/oncotarget.17093. PMID: 28467774 PMC5564749

[40] Ren L Zhou T Wang Y Wu Y Xu H Liu J . RNF8 induces β-catenin-mediated c-Myc expression and promotes colon cancer proliferation. Int J Biol Sci. (2020) 16:2051–62. doi: 10.7150/ijbs.44119. PMID: 32549753 PMC7294952

[41] Shang S Hua F Hu ZW . The regulation of β-catenin activity and function in cancer: therapeutic opportunities. Oncotarget. (2017) 8:33972–89. doi: 10.18632/oncotarget.15687. PMID: 28430641 PMC5464927

[42] Behrouj H Mokarram P . BAMLET (Bovine α-lactalbumin made lethal to tumor cells) inhibits autophagy flux and induces apoptosis via down-regulation of protein kinase CK1α and attenuation of the AKT/p-ß-catenin (S552) pathway in RAS-mutated human colorectal HCT 116 cells. Iran J Basic. Med Sci. (2023) 26:1212–9. doi: 10.22038/IJBMS.2023.69343.15114. PMID: 37736507 PMC10510486

[43] Liang J Sun L Li Y Liu W Li D Chen P . Wnt signaling modulator DKK4 inhibits colorectal cancer metastasis through an AKT/Wnt/β-catenin negative feedback pathway. J Biol Chem. (2022) 298:102545. doi: 10.1016/j.jbc.2022.102545. PMID: 36181792 PMC9640985

[44] Fleming-de-Moraes CD Rocha MR Tessmann JW de Araujo WM Morgado-Diaz JA . Crosstalk between PI3K/Akt and Wnt/β-catenin pathways promote colorectal cancer progression regardless of mutational status. Cancer Biol Ther. (2022) 23:1–13. doi: 10.1080/15384047.2022.2108690. PMID: 35944058 PMC9367664

[45] Lezcano V Gentili C de Boland AR . Role of PTHrP in human intestinal Caco-2 cell response to oxidative stress. Biochim Biophys Acta. (2013) 1833:2834–43. doi: 10.1016/j.bbamcr.2013.06.029. PMID: 23845990

[46] Wang C Yan J Yin P Gui L Ji L Ma B . β-catenin inhibition shapes tumor immunity and synergizes with immunotherapy in colorectal cancer. Oncoimmunology. (2020) 9:1809947. doi: 10.1080/2162402X.2020.1809947. PMID: 32939327 PMC7470182

[47] Arango D Wilson AJ Shi Q Corner GA Arañes MJ Nicholas C . Molecular mechanisms of action and prediction of response to oxaliplatin in colorectal cancer cells. Br J Cancer. (2004) 91:1931–46. doi: 10.1038/sj.bjc.6602215. PMID: 15545975 PMC2409767

[48] Alsina-Sanchis E Mülfarth R Fischer A . Control of tumor progression by angiocrine factors. Cancers. (2021) 13:2610. doi: 10.3390/cancers13112610. PMID: 34073394 PMC8198241

[49] Maishi N Annan DA Kikuchi H Hida Y Hida K . Tumor endothelial heterogeneity in cancer progression. Cancers. (2019) 11:1511. doi: 10.1016/j.ccell.2019.08.007. PMID: 31600937 PMC6826555

[50] Garona J Sobol NT Pifano M Segatori VI Gomez DE Ripoll GV . Preclinical efficacy of [V4 Q5] dDAVP, a second generation vasopressin analog, on metastatic spread and tumor-associated angiogenesis in colorectal cancer. Cancer Res Treat. (2019) 51:438–50. doi: 10.4143/crt.2018.040. PMID: 29879760 PMC6473275

[51] Sato Y Fu Y Liu H Lee M Shaw M . Tumor-immune profiling of CT-26 and Colon 26 syngeneic mouse models reveals mechanism of anti-PD-1 response. BMC Cancer. (2021) 21:1222. doi: 10.1186/s12885-021-08974-3. PMID: 34774008 PMC8590766

[52] Chai WX Sun LG Dai FH Shao HS Zheng NG Cai HY . Inhibition of PRRX2 suppressed colon cancer liver metastasis via inactivation of Wnt/β-catenin signaling pathway. Pathol Res Pract. (2019) 215:152593. doi: 10.1016/j.prp.2019.152593. PMID: 31471104

[53] Huang X Lao X He C Wang J Pan Y . The mechanism of sevoflurane affecting ovarian cancer cell proliferation and migration by regulating RNA methylase TRDMT1 to activate the β-catenin pathway. Cell Biol Toxicol. (2024) 40:108. doi: 10.1007/s10565-024-09941-x. PMID: 39630363 PMC11618209

[54] Luo ZD Wang YF Zhao YX Yu LC Li T Fan YJ . Emerging roles of non-coding RNAs in colorectal cancer oxaliplatin resistance and liquid biopsy potential. World J Gastroenterol. (2023) 29(1):1–18. doi: 10.3748/wjg.v29.i1.1 PMC9850945 36683709

[55] Ryu K Seo J Lee K Choi J Yoo G Ha J . Drug-resistance biomarkers in patient-derived colorectal cancer organoid and fibroblast co-culture system. Curr Issues Mol Biol. (2024) 46:5794–811. doi: 10.3390/cimb46060346. PMID: 38921017 PMC11202770

[56] Linares J Sallent-Aragay A Badia-Ramentol J Recort-Bascuas A Méndez A Manero-Rupérez N . Long-term platinum-based drug accumulation in cancer-associated fibroblasts promotes colorectal cancer progression and resistance to therapy. Nat Commun. (2023) 14:746. doi: 10.1038/s41467-023-36334-1. PMID: 36765091 PMC9918738

[57] Zhang H Yu C Dai J Keller JM Hua A Sottnik JL . Parathyroid hormone-related protein inhibits DKK1 expression through c-Jun-mediated inhibition of β-catenin activation of the DKK1 promoter in prostate cancer. Oncogene. (2014) 33:2464–77. doi: 10.1038/onc.2013.203. PMID: 23752183 PMC4004708

[58] Aktary Z Bertrand JU Larue L . The WNT-less wonder: WNT-independent β-catenin signaling. Pigment. Cell Melanoma. Res. (2016) 29:524–40. doi: 10.1111/pcmr.12501. PMID: 27311806

[59] Wang J Han L Sinnett-Smith J Han LL Stevens JV Rozengurt N . Positive cross talk between protein kinase D and β-catenin in intestinal epithelial cells: impact on β-catenin nuclear localization and phosphorylation at Ser552. Am J Physiol Am J Physiol Cell Physiol. (2016) 310:C542–57. doi: 10.1152/ajpcell.00302.2015. PMID: 26739494 PMC4824155

[60] Xu H Xie X Han J Zhang C . miR-340 reverses chemotherapy resistance in colon cancer via the PDCD4/WNT/β-catenin signalling pathway. Sci Rep. (2025) 15(1):33421. doi: 10.1038/s41598-025-18832-y 41022995 PMC12480034

[61] Huang Y Chan S Chen S Liu X Li M Zheng L . Wnt/β-catenin signalling activates IMPDH2-mediated purine metabolism to facilitate oxaliplatin resistance by inhibiting caspase-dependent apoptosis in colorectal cancer. J Transl Med. (2024) 22:133. doi: 10.1186/s12967-024-04934-0. PMID: 38310229 PMC10838440

[62] Yu PC Liu D Han ZX Liang F Hao CY Lei YT . Thymopentin-mediated inhibition of cancer stem cell stemness enhances the cytotoxic effect of oxaliplatin on colon cancer cells. Front Pharmacol. (2022) 13:779715. doi: 10.3389/fphar.2022.779715. PMID: 35242031 PMC8886222

[63] Guturi KKN Mandal T Chatterjee A Sarkar M Bhattacharya S Chatterjee U . Mechanism of β-catenin-mediated transcriptional regulation of epidermal growth factor receptor expression in glycogen synthase kinase 3 β-inactivated prostate cancer cells. J Biol Chem. (2012) 287:18287–96. doi: 10.1074/jbc.M111.324798. PMID: 22493441 PMC3365735

[64] Zhao J Yue W Zhu MJ Sreejayan N Du M . AMP-activated protein kinase (AMPK) cross-talks with canonical Wnt signaling via phosphorylation of beta-catenin at Ser 552. Biochem Biophys Res Commun. (2010) 395:146–51. doi: 10.1016/j.bbrc.2010.03.161. PMID: 20361929 PMC2864303

[65] Trujano-Camacho S Cantú-de León D Delgado-Waldo I Coronel-Hernández J Millan-Catalan O Hernández-Sotelo . Inhibition of Wnt-β-catenin signaling by ICRT14 drug depends of post-transcriptional regulation by HOTAIR in human cervical cancer HeLa cells. Front Oncol. (2021) 11:729228. doi: 10.3389/fonc.2021.729228. PMID: 34778043 PMC8580948

[66] Du J Liu H Mao X Qin Y Fan C . ATF4 promotes lung cancer cell proliferation and invasion partially through regulating Wnt/β-catenin signaling. BioMed Pharmacother. (2021) 168:115663. doi: 10.1016/j.biopha.2023.115663. PMID: 33628101 PMC7893563

[67] Mezquita B Pineda E Mezquita J Mezquita P Pau M Codony-Servat J . LoVo colon cancer cells resistant to oxaliplatin overexpress c-MET and VEGFR-1 and respond to VEGF with dephosphorylation of c-MET. Mol Carcinog. (2016) 55:411–9. doi: 10.1002/mc.22289. PMID: 25647613

[68] Wang P Sun L Li C Jin B Yang H Wu B . Study on drug screening multicellular model for colorectal cancer constructed by three-dimensional bioprinting technology. Int J Bioprint. (2023) 9:694. doi: 10.18063/ijb.694. PMID: 37273979 PMC10236483

[69] Lu Y Xiao G Galson DL Nishio Y Mizokami A Keller ET . PTHrP-induced MCP-1 production by human bone marrow endothelial cells and osteoblasts promotes osteoclast differentiation and prostate cancer cell proliferation and invasion *in vitro*. Int J Cancer. (2007) 121:724–33. doi: 10.1002/ijc.22704. PMID: 17390372

[70] Wilczyński B Dąbrowska A Kulbacka J Baczyńska D . Chemoresistance and the tumor microenvironment: the critical role of cell-cell communication. Cell Commun Signal. (2024) 22:486. doi: 10.1186/s12964-024-01857-7. PMID: 39390572 PMC11468187

[71] Choi YJ Kim JH Rho JK Kim JS Choi CM Kim WS . AXL and MET receptor tyrosine kinases are essential for lung cancer metastasis. Oncol Rep. (2017) 37:2201–8. doi: 10.3892/or.2017.5482. PMID: 28260071

[72] Purcell R Childs M Maibach R Miles C Turner C Zimmermann A . HGF/c-Met related activation of β-catenin in hepatoblastoma. J Exp Clin Cancer Res. (2011) 30:96. doi: 10.1186/1756-9966-30-96. PMID: 21992464 PMC3207961

[73] Herynk MH Tsan R Radinsky R Gallick GE . Activation of c-Met in colorectal carcinoma cells leads to constitutive association of tyrosine-phosphorylated beta-catenin. Clin Exp Metastasis. (2003) 20:291–300. doi: 10.1023/a:1024024218529. PMID: 12856716

[74] Lee GT Rosenfeld JA Kim WT Kwon YS Palapattu G Mehra R . TCF4 induces enzalutamide resistance via neuroendocrine differentiation in prostate cancer. PloS One. (2019) 14:e0213488. doi: 10.1371/journal.pone.0213488. PMID: 31536510 PMC6752758

[75] Panaampon J Sasamoto K Kariya R Okada S . Establishment of nude mice lacking NK cells and their application for human tumor xenografts. Asian Pac. J Cancer Prev. (2021) 22:1069–74. doi: 10.31557/APJCP.2021.22.4.1069. PMID: 33906298 PMC8325116

[76] Jang SH Wientjes MG Lu D Au JL-S . Drug delivery and transport to solid tumors. Pharm Res. (2003) 20:1337–50. doi: 10.1023/A:1025785505977. PMID: 14567626

[77] Au JL-S Jang SH Zheng J Chen C-T Song S Hu L . Determinants of drug delivery and transport to solid tumors. J Ctrl. Release. (2001) 74:31–46. doi: 10.1016/S0168-3659(01)00308-X. PMID: 11489481

[78] Momin N Palmeri JR Lutz EA Jailkhani N Mak H Tabet A . Maximizing response to intratumoral immunotherapy in mice by tuning local retention. Nat Commun. (2022) 13:109. doi: 10.1038/s41467-021-27390-6. PMID: 35013154 PMC8748612

[79] Kienzl M Maitz K Sarsembayeva A Valadez-Cosmes P Gruden E Ristic D . Comparative study of the immune microenvironment in heterotopic tumor models. Cancers. (2024) 16:295. doi: 10.3390/cancers16020295. PMID: 38254785 PMC10813609

[80] Yin Y Yao S Hu Y Feng Y Li M Bian Z . The immune-microenvironment confers chemoresistance of colorectal cancer through macrophage-derived IL6. Clin Cancer Res. (2017) 23:7375–87. doi: 10.1158/1078-0432.CCR-17-1283. PMID: 28928161

[81] Funk JL Krul EJ Moser AH Shigenaga JK Strewler GJ Grunfeld C . Endotoxin increases parathyroid hormone-related protein mRNA levels in mouse spleen. Mediation by tumor necrosis factor. J Clin Invest. (1993) 92:2546–52. doi: 10.1172/JCI116864. PMID: 8227368 PMC288441

[82] Taurin S Sandbo N Qin Y Browning D Dulin NO . Phosphorylation of beta-catenin by cyclic AMP-dependent protein kinase. J Biol Chem. (2006) 281(15):9971–6. doi: 10.1074/jbc.M508778200 16476742

