# Supplementary material for: Study of ß-catenin signaling pathway modulated by PTHrP in cell and animal colorectal cancer models
Source: Front Oncol. 2026 May 29;16:1773635. doi: 10.3389/fonc.2026.1773635 (PMC13259664; doi:10.3389/fonc.2026.1773635)
Supplement: Supplementary file 1 [file DataSheet1.pdf]

Supplementary Material

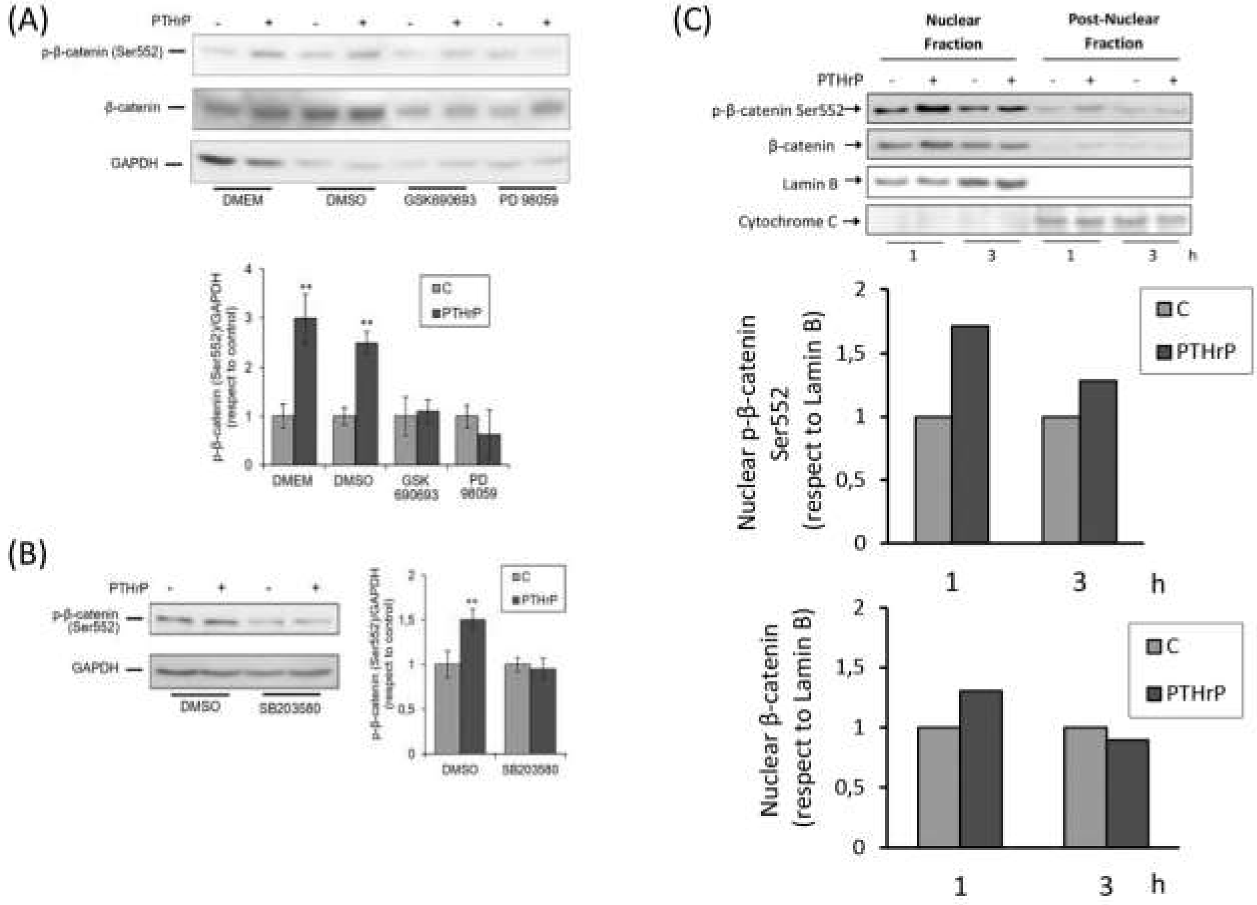

PTHrP promotes the nuclear localization of p-β-catenin (Ser552). A. Akt, ERK MAPK and **B.** p38 MAPK pathways modulate PTHrP-induced phosphorylation of β-catenin. CRC cells were pre-incubated with **A.** GSK690693 (50 nmol/L, Akt inhibitor), PD98059 (20mmol/L, ERK 1/2 MAPK inhibitor) or **B.** SB203580 (20 µmol/L, p38 MAPK inhibitor) for 30 minutes and then treated with PTHrP 10^{-8} mol/L for 1 hour. Proteins from lysates were processed for Western blot assay, separated on SDS-PAGE and immunoblots were performed using anti-p-β-catenin (Ser552) antibody. The antibody against GAPDH was used to assess the equivalence of protein content. ** p <0.01. **C.** CRC cells were treated with PTHrP 10^{-8} mol/L for 1 to 3 hours and then the nuclear and post-nuclear fractions were separated by cellular fractionation. To confirm the purity of the fractions and the amount of protein, antibodies that recognize the nuclear protein Lamin B, and the cytosolic protein cytochrome C were used. DMEM: Dulbecco's Modified Eagle Medium. DMSO: dimethylsulfoxide.
